# Supplementary material for: Variant-specific symptoms of COVID-19 in a study of 1,542,510 adults in England
Source: Nat Commun. 2022 Nov 11;13:6856. doi: 10.1038/s41467-022-34244-2 (PMC9651890; doi:10.1038/s41467-022-34244-2)
Supplement: Supplementary file 1 — Supplementary Information [file 41467_2022_34244_MOESM1_ESM.pdf]

Variant-specific symptoms of COVID-19 in a study of 1,542,510 adults in England: Supplementary material

Matthew Whitaker, Joshua Elliott, Barbara Bodinier, Wendy Barclay, Helen Ward, Graham Cooke, Christl A. Donnelly, Marc Chadeau-Hyam, Paul Elliott

Supplementary tables

Supplementary Table 1 Characteristics of the study population.
Supplementary Table 2 Characteristics of swab positives in the study population.
Supplementary Table 3 Odds ratios for swab positivity based on presence or absence of any of 26 symptoms surveyed across selected rounds of REACT-1.
Supplementary Table 4 Unadjusted odds ratios for swab positivity based on presence or absence of any of 26 symptoms surveyed across selected rounds of REACT-1
Supplementary Table 5 Results from log-linear regression of symptom count as a function of multiple covariates.

Supplementary figures

Supplementary Figure 1 Comparison of Ct values in swab-positive respondents with Omicron BA.1 and BA.2 variants in REACT-1 rounds 17–19, stratified by symptom status (presence of any of 26 surveyed symptoms).
Supplementary Figure 2 Plot showing the odds ratios and 95% confidence intervals for symptoms in a pooled analysis of N=1,542,510 participants across five variant-phases.
Supplementary Figure 3 Results of LASSO stability selection with swab positive/negative as the binary outcome variable and each of 26 symptoms as predictors, in five phases of REACT-1 corresponding to periods of dominance of five SARS-CoV-2 variants in England.
Supplementary Figure 4 Heatmaps showing co-occurrence of symptoms across variants among swab-positive individuals.
Supplementary Figure 5 Heatmap showing co-occurrence of first symptoms among swab positive individuals in rounds 17–19, when Omicron was dominant.
Supplementary Figure 6 Scatter plot showing N-gene Ct values among symptomatic swab-positive individuals plotted against time (in days) since symptom onset, by BA.1/BA.2.
Supplementary Figure 7 Panel plot showing time since symptom onset across different variant phases
Supplementary Figure 8 Panel plot exploring Ct values and PCR positivity rates among those who were excluded from analysis owing to missing symptom data.
Supplementary Figure 9 Prevalence of any of 26 symptoms in PCR negative vs PCR positive individuals in each round of REACT-1 between June 2020 and March 2022.

Supplementary methods

Survey symptom questions
Data exclusions
Model specifications
Assessing model performance in variable selection

Supplementary Table 1 Characteristics of study population. N (percent [95% CI]) or mean (SD).

Variable	Category	Rounds 2-7 (Wild-type)	Rounds 8-10 (Alpha)	Rounds 13-15 (Delta)	Rounds 17-19 (Omicron)	Sum or mean(SD)
All participants	All participants	713,202 (100%, [100-100])	357,960 (100%, [100-100])	228,934 (100%, [100-100])	242,414 (100%, [100-100])	1,542,510
Sex	Female	402,121 (56.4%, [56.3-56.5])	201,126 (56.2%, [56-56.3])	129,652 (56.6%, [56.4-56.8])	139,341 (57.5%, [57.3-57.7])	872,240
	Male	311,081 (43.6%, [43.5-43.7])	156,834 (43.8%, [43.7-44])	99,282 (43.4%, [43.2-43.6])	103,073 (42.5%, [42.3-42.7])	670,270
Age group	18-24	32,298 (4.5%, [4.5-4.6])	15,805 (4.4%, [4.3-4.5])	7,268 (3.2%, [3.1-3.2])	15,662 (6.5%, [6.4-6.6])	71,033
	25-34	73,352 (10.3%, [10.2-10.4])	35,260 (9.9%, [9.8-9.9])	19,691 (8.6%, [8.5-8.7])	34,242 (14.1%, [14-14.3])	162,545
	35-44	103,317 (14.5%, [14.4-14.6])	51,269 (14.3%, [14.2-14.4])	30,996 (13.5%, [13.4-13.7])	37,425 (15.4%, [15.3-15.6])	223,007
	45-54	137,175 (19.2%, [19.1-19.3])	68,057 (19%, [18.9-19.1])	43,205 (18.9%, [18.7-19])	37,937 (15.6%, [15.5-15.8])	286,374
	55-64	157,180 (22%, [21.9-22.1])	80,550 (22.5%, [22.4-22.6])	53,663 (23.4%, [23.3-23.6])	48,896 (20.2%, [20-20.3])	340,289
	65-74	144,612 (20.3%, [20.2-20.4])	74,535 (20.8%, [20.7-21])	50,980 (22.3%, [22.1-22.4])	45,876 (18.9%, [18.8-19.1])	316,003
	75+	65,268 (9.2%, [9.1-9.2])	32,484 (9.1%, [9-9.2])	23,131 (10.1%, [10-10.2])	22,376 (9.2%, [9.1-9.3])	143,259
Ethnicity	Asian	25,317 (3.6%, [3.6-3.6])	11,665 (3.3%, [3.3-3.4])	10,202 (4.6%, [4.5-4.6])	13,192 (5.6%, [5.5-5.7])	60,376
	Black	6,736 (1%, [0.9-1])	3,497 (1%, [1-1])	3,441 (1.5%, [1.5-1.6])	3,820 (1.6%, [1.6-1.7])	17,494
	Mixed	8,206 (1.2%, [1.1-1.2])	4,141 (1.2%, [1.1-1.2])	2,886 (1.3%, [1.2-1.3])	3,554 (1.5%, [1.5-1.6])	18,787
	Other	5,066 (0.7%, [0.7-0.7])	2,382 (0.7%, [0.7-0.7])	2,082 (0.9%, [0.9-1])	2,232 (0.9%, [0.9-1])	11,762
	White	658,752 (93.6%, [93.5-93.6])	330,027 (93.8%, [93.8-93.9])	205,368 (91.7%, [91.6-91.8])	213,938 (90.4%, [90.3-90.5])	1,408,085
PCR positive at time of survey	No	710,231 (99.6%, [99.6-99.6])	355,685 (99.4%, [99.3-99.4])	227,441 (99.3%, [99.3-99.4])	231,705 (95.6%, [95.5-95.7])	1,525,062
	Yes	2,971 (0.4%, [0.4-0.4])	2,275 (0.6%, [0.6-0.7])	1,493 (0.7%, [0.6-0.7])	10,709 (4.4%, [4.3-4.5])	17,448
Prior COVID-19	No previous COVID-19	604,198 (84.7%, [84.6-84.8])	301,856 (84.3%, [84.2-84.4])	189,854 (82.9%, [82.8-83.1])	165,113 (68.1%, [67.9-68.3])	1,261,021
	Previous COVID-19 greater than 28 days	3,308 (0.5%, [0.4-0.5])	10,374 (2.9%, [2.8-3])	14,428 (6.3%, [6.2-6.4])	42,367 (17.5%, [17.3-17.6])	70,477
	Previous COVID-19 with unknown time	26 (0%, [0-0])	56 (0%, [0-0])	34 (0%, [0-0])	67 (0%, [0-0])	183
	Previous COVID-19 within 28 days	1,276 (0.2%, [0.2-0.2])	1,906 (0.5%, [0.5-0.6])	1,659 (0.7%, [0.7-0.8])	13,349 (5.5%, [5.4-5.6])	18,190
	Suspected previous COVID-19	103,703 (14.5%, [14.5-14.6])	43,483 (12.1%, [12-12.3])	22,805 (10%, [9.8-10.1])	21,333 (8.8%, [8.7-8.9])	191,324

	Unknown	691 (0.1%, [0.1-0.1])	285 (0.1%, [0.1-0.1])	154 (0.1%, [0.1-0.1])	185 (0.1%, [0.1-0.1])	1,315
Vaccination status	NA	-	-	912 (1.2%, [1.1-1.2])	2,882 (1.2%, [1.1-1.2])	3,794
	Not vaccinated	-	-	3,650 (4.6%, [4.5-4.8])	9,030 (3.7%, [3.7-3.8])	12,680
	One dose	-	-	519 (0.7%, [0.6-0.7])	1,496 (0.6%, [0.6-0.6])	2,015
	Three doses	-	-	14,388 (18.2%, [17.9-18.5])	198,035 (81.7%, [81.5-81.8])	212,423
	Two doses	-	-	56,135 (71%, [70.7-71.3])	20,246 (8.4%, [8.2-8.5])	76,381
	Unknown doses	-	-	3,433 (4.3%, [4.2-4.5])	10,725 (4.4%, [4.3-4.5])	14,158
Symptoms	Symptomatic	95,973 (13.5%, [13.4-13.5])	49,228 (13.8%, [13.6-13.9])	36,687 (16%, [15.9-16.2])	52,983 (21.9%, [21.7-22])	234,871
Number of reported symptoms	Mean (SD)	0.52 (1.66)	0.5 (1.63)	0.61 (1.84)	0.91 (2.34)	0.6 (1.83)
Sought medical attention for symptoms	No	87,252 (89.5%, [89.3-89.7])	43,636 (88.9%, [88.6-89.2])	31,933 (87.2%, [86.8-87.5])	47,231 (89.2%, [89-89.5])	210,052
	Yes	10,203 (10.5%, [10.3-10.7])	5,457 (11.1%, [10.8-11.4])	4,701 (12.8%, [12.5-13.2])	5,698 (10.8%, [10.5-11])	26,059
Symptoms affecting day-to-day activities	A lot	-	-	2,291 (1%, [1-1])	4,931 (2%, [2-2.1])	7,222
	A little	-	-	7,849 (3.4%, [3.4-3.5])	15,314 (6.3%, [6.2-6.4])	23,163
	Not at all	-	-	4,938 (2.2%, [2.1-2.2])	9,270 (3.8%, [3.7-3.9])	14,208
	Don't know / PNA / Non-response	-	-	21,609 (9.4%, [9.3-9.6])	23,468 (9.7%, [9.6-9.8])	190,278
	No symptoms reported	-	-	192,247 (84%, [83.8-84.1])	189,431 (78.1%, [78-78.3])	1,307,639
Days since onset of first symptom	Mean (SD)	7.93 (3.28)	8.09 (3.28)	7.89 (3.32)	7.97 (3.29)	7.95 (3.29)
Symptom in past 7 days	Loss or change of sense of smell	2,453 (0.3%, [0.3-0.4])	1,392 (0.4%, [0.4-0.4])	1,460 (0.6%, [0.6-0.7])	2,273 (0.9%, [0.9-1])	7,578
	Loss or change of sense of taste	2,876 (0.4%, [0.4-0.4])	1,551 (0.4%, [0.4-0.5])	1,420 (0.6%, [0.6-0.7])	2,362 (1%, [0.9-1])	8,209
	New persistent cough	4,710 (0.7%, [0.6-0.7])	2,120 (0.6%, [0.6-0.6])	3,599 (1.6%, [1.5-1.6])	7,212 (3%, [2.9-3])	17,641
	Fever	5,666 (0.8%, [0.8-0.8])	2,515 (0.7%, [0.7-0.7])	2,619 (1.1%, [1.1-1.2])	4,450 (1.8%, [1.8-1.9])	15,250
	Runny nose	24,262 (3.4%, [3.4-3.4])	12,008 (3.4%, [3.3-3.4])	11,105 (4.9%, [4.8-4.9])	19,335 (8%, [7.9-8.1])	66,710
	Sneezing	24,496 (3.4%, [3.4-3.5])	10,909 (3%, [3-3.1])	9,673 (4.2%, [4.1-4.3])	15,087 (6.2%, [6.1-6.3])	60,165
	Blocked nose	17,806 (2.5%, [2.5-2.5])	8,504 (2.4%, [2.3-2.4])	8,067 (3.5%, [3.4-3.6])	13,507 (5.6%, [5.5-5.7])	47,884

Sore eyes	14,341 (2%, [2-2])	6,777 (1.9%, [1.8-1.9])	4,628 (2%, [2-2.1])	7,038 (2.9%, [2.8-3])	32,784
Sore throat	27,295 (3.8%, [3.8-3.9])	10,259 (2.9%, [2.8-2.9])	10,410 (4.5%, [4.5-4.6])	15,571 (6.4%, [6.3-6.5])	63,535
Hoarse voice	8,322 (1.2%, [1.1-1.2])	3,292 (0.9%, [0.9-1])	4,509 (2%, [1.9-2])	7,247 (3%, [2.9-3.1])	23,370
Headache	39,332 (5.5%, [5.5-5.6])	20,835 (5.8%, [5.7-5.9])	13,727 (6%, [5.9-6.1])	21,475 (8.9%, [8.7-9])	95,369
Dizziness	12,857 (1.8%, [1.8-1.8])	6,128 (1.7%, [1.7-1.8])	4,372 (1.9%, [1.9-2])	6,307 (2.6%, [2.5-2.7])	29,664
Appetite loss	6,676 (0.9%, [0.9-1])	3,318 (0.9%, [0.9-1])	2,736 (1.2%, [1.2-1.2])	4,296 (1.8%, [1.7-1.8])	17,026
Nausea/vomiting	8,185 (1.1%, [1.1-1.2])	3,884 (1.1%, [1.1-1.1])	2,825 (1.2%, [1.2-1.3])	3,948 (1.6%, [1.6-1.7])	18,842
Diarrhoea	11,107 (1.6%, [1.5-1.6])	4,799 (1.3%, [1.3-1.4])	3,573 (1.6%, [1.5-1.6])	4,741 (2%, [1.9-2])	24,220
Abdominal pain / belly ache	14,707 (2.1%, [2-2.1])	6,934 (1.9%, [1.9-2])	4,619 (2%, [2-2.1])	6,096 (2.5%, [2.5-2.6])	32,356
Shortness of breath	12,907 (1.8%, [1.8-1.8])	6,634 (1.9%, [1.8-1.9])	4,587 (2%, [1.9-2.1])	7,647 (3.2%, [3.1-3.2])	31,775
Tight chest	8,977 (1.3%, [1.2-1.3])	4,514 (1.3%, [1.2-1.3])	2,969 (1.3%, [1.3-1.3])	4,736 (2%, [1.9-2])	21,196
Chest pain	4,158 (0.6%, [0.6-0.6])	2,279 (0.6%, [0.6-0.7])	1,529 (0.7%, [0.6-0.7])	2,494 (1%, [1-1.1])	10,460
Chills	7,077 (1%, [1-1])	5,429 (1.5%, [1.5-1.6])	2,652 (1.2%, [1.1-1.2])	5,955 (2.5%, [2.4-2.5])	21,113
Difficulty sleeping	26,551 (3.7%, [3.7-3.8])	14,043 (3.9%, [3.9-4])	8,956 (3.9%, [3.8-4])	13,035 (5.4%, [5.3-5.5])	62,585
Tiredness	39,897 (5.6%, [5.5-5.6])	19,756 (5.5%, [5.4-5.6])	14,813 (6.5%, [6.4-6.6])	23,072 (9.5%, [9.4-9.6])	97,538
Severe fatigue	4,677 (0.7%, [0.6-0.7])	2,715 (0.8%, [0.7-0.8])	2,063 (0.9%, [0.9-0.9])	3,482 (1.4%, [1.4-1.5])	12,937
Numbness/tingling	9,748 (1.4%, [1.3-1.4])	4,725 (1.3%, [1.3-1.4])	3,214 (1.4%, [1.4-1.5])	4,295 (1.8%, [1.7-1.8])	21,982
Heavy arms/legs	8,527 (1.2%, [1.2-1.2])	4,017 (1.1%, [1.1-1.2])	2,707 (1.2%, [1.1-1.2])	4,080 (1.7%, [1.6-1.7])	19,331
Muscle aches	21,342 (3%, [3-3])	11,069 (3.1%, [3-3.1])	7,488 (3.3%, [3.2-3.3])	11,866 (4.9%, [4.8-5])	51,765

Supplementary Table 2 Characteristics of swab positives in study population. N (percent [95% CI]) or mean (SD). NOTE: Individuals with Wild-type, Alpha and Delta variants were inferred by the time of infection; those with BA.2 and BA.1 variants were identified using sequencing. For this reason, the sum total of PCR positives with Omicron is lower in this table than the total reported in Supplementary Table 1, with the difference being those who either were not sequenced from rounds 17–19, or those who were sequenced but did not have BA.2 or BA.1.

Variable	Category	Rounds 2-7 (Wild-type)	Rounds 8-10 (Alpha)	Rounds 12-15 (Delta)	BA.1 (Omicron)	BA.2 (Omicron)	Total
All participants	All participants	2,971 (100%, [99.9-100])	2,275 (100%, [99.9-100])	1,493 (100%, [99.8-100])	2,835 (100%, [99.9-100])	3,560 (100%, [99.9-100])	13,134
Sex	Female	1,683 (56.6%, [54.9-58.4])	1,283 (56.4%, [54.3-58.4])	809 (54.2%, [51.7-56.7])	1,617 (57%, [55.2-58.8])	2,043 (57.4%, [55.8-59])	7,435
	Male	1,288 (43.4%, [41.6-45.1])	992 (43.6%, [41.6-45.7])	684 (45.8%, [43.3-48.3])	1,218 (43%, [41.2-44.8])	1,517 (42.6%, [41-44.2])	5,699
Age group	18-24	239 (8%, [7.1-9.1])	178 (7.8%, [6.8-9])	60 (4%, [3.1-5.1])	153 (5.4%, [4.6-6.3])	235 (6.6%, [5.8-7.5])	865
	25-34	401 (13.5%, [12.3-14.8])	289 (12.7%, [11.4-14.1])	125 (8.4%, [7.1-9.9])	389 (13.7%, [12.5-15])	665 (18.7%, [17.4-20])	1,869
	35-44	475 (16%, [14.7-17.3])	401 (17.6%, [16.1-19.2])	298 (20%, [18-22.1])	484 (17.1%, [15.7-18.5])	688 (19.3%, [18.1-20.7])	2,346
	45-54	642 (21.6%, [20.2-23.1])	493 (21.7%, [20-23.4])	371 (24.8%, [22.7-27.1])	541 (19.1%, [17.7-20.6])	555 (15.6%, [14.4-16.8])	2,602
	55-64	618 (20.8%, [19.4-22.3])	510 (22.4%, [20.8-24.2])	307 (20.6%, [18.6-22.7])	618 (21.8%, [20.3-23.4])	646 (18.1%, [16.9-19.4])	2,699
	65-74	429 (14.4%, [13.2-15.7])	299 (13.1%, [11.8-14.6])	247 (16.5%, [14.7-18.5])	471 (16.6%, [15.3-18])	554 (15.6%, [14.4-16.8])	2,000
	75+	167 (5.6%, [4.8-6.5])	105 (4.6%, [3.8-5.6])	85 (5.7%, [4.6-7])	179 (6.3%, [5.5-7.3])	217 (6.1%, [5.4-6.9])	753
Ethnicity	Asian	158 (5.4%, [4.6-6.3])	137 (6.1%, [5.2-7.2])	84 (5.7%, [4.6-7])	137 (4.9%, [4.2-5.8])	173 (5%, [4.3-5.7])	689
	Black	40 (1.4%, [1-1.9])	42 (1.9%, [1.4-2.5])	31 (2.1%, [1.5-3])	49 (1.8%, [1.3-2.3])	45 (1.3%, [1-1.7])	207
	Mixed	26 (0.9%, [0.6-1.3])	30 (1.3%, [0.9-1.9])	14 (1%, [0.6-1.6])	50 (1.8%, [1.4-2.4])	73 (2.1%, [1.7-2.6])	193
	Other	23 (0.8%, [0.5-1.2])	20 (0.9%, [0.6-1.4])	19 (1.3%, [0.8-2])	26 (0.9%, [0.6-1.4])	37 (1.1%, [0.8-1.5])	125
	White	2,676 (91.5%, [90.5-92.5])	2,002 (89.7%, [88.4-90.9])	1,319 (89.9%, [88.3-91.3])	2,510 (90.5%, [89.4-91.6])	3,149 (90.6%, [89.5-91.5])	11,656
PCR positive at time of survey	Yes	2,971 (100%, [99.9-100])	2,275 (100%, [99.9-100])	1,493 (100%, [99.8-100])	2,835 (100%, [99.9-100])	3,560 (100%, [99.9-100])	13,134
Prior COVID-19	No previous COVID-19	1,840 (61.9%, [60.2-63.7])	1,011 (44.4%, [42.4-46.5])	563 (37.7%, [35.3-40.2])	816 (28.8%, [27.1-30.5])	877 (24.6%, [23.2-26.1])	5,107
	Previous COVID-19 greater than 28 days	35 (1.2%, [0.8-1.6])	86 (3.8%, [3.1-4.6])	51 (3.4%, [2.6-4.5])	148 (5.2%, [4.5-6.1])	198 (5.6%, [4.9-6.4])	518
	Previous COVID-19 with unknown time	3 (0.1%, [0-0.3])	4 (0.2%, [0.1-0.5])	0 (0%, [0-0.2])	2 (0.1%, [0-0.3])	1 (0%, [0-0.2])	10

	Previous COVID-19 within 28 days	586 (19.7%, [18.3-21.2])	766 (33.7%, [31.8-35.6])	722 (48.4%, [45.8-50.9])	1,665 (58.7%, [56.9-60.5])	2,188 (61.5%, [59.9-63])	5,927
	Suspected previous COVID-19	500 (16.8%, [15.5-18.2])	403 (17.7%, [16.2-19.3])	152 (10.2%, [8.7-11.8])	200 (7.1%, [6.2-8.1])	291 (8.2%, [7.3-9.1])	1,546
	Unknown	7 (0.2%, [0.1-0.5])	5 (0.2%, [0.1-0.5])	5 (0.3%, [0.1-0.8])	4 (0.1%, [0.1-0.4])	5 (0.1%, [0.1-0.3])	26
Vaccination status	NA	-	-	11 (1.5%, [0.8-2.7])	36 (1.3%, [0.9-1.8])	33 (0.9%, [0.7-1.3])	80
	Not vaccinated	-	-	28 (3.8%, [2.6-5.4])	77 (2.7%, [2.2-3.4])	106 (3%, [2.5-3.6])	211
	One dose	-	-	11 (1.5%, [0.8-2.7])	15 (0.5%, [0.3-0.9])	23 (0.6%, [0.4-1])	49
	Three doses	-	-	73 (9.9%, [8-12.3])	2,276 (80.3%, [78.8-81.7])	2,925 (82.2%, [80.9-83.4])	5,274
	Two doses	-	-	579 (78.6%, [75.5-81.4])	284 (10%, [9-11.2])	265 (7.4%, [6.6-8.4])	1,128
	Unknown doses	-	-	35 (4.7%, [3.4-6.5])	147 (5.2%, [4.4-6.1])	208 (5.8%, [5.1-6.7])	390
Symptoms	Symptomatic	1,338 (45%, [43.3-46.8])	1,245 (54.7%, [52.7-56.8])	952 (63.8%, [61.3-66.2])	1,984 (70%, [68.3-71.6])	2,701 (75.9%, [74.4-77.2])	8,220
Number of reported symptoms	Mean (SD)	2.7 (4.1)	3.38 (4.5)	4.63 (5.21)	4.63 (4.72)	6.02 (5.26)	4.15 (4.83)
Sought medical attention for symptoms	No	1,187 (88.7%, [86.9-90.3])	1,091 (87.7%, [85.8-89.4])	850 (89.5%, [87.4-91.3])	1,865 (94.1%, [93-95.1])	2,522 (93.4%, [92.4-94.3])	7,515
	Yes	151 (11.3%, [9.7-13.1])	153 (12.3%, [10.6-14.2])	100 (10.5%, [8.7-12.6])	117 (5.9%, [4.9-7])	179 (6.6%, [5.7-7.6])	700
Symptoms affecting day-to-day activities	A lot	-	-	157 (10.5%, [9.1-12.2])	303 (10.7%, [9.6-11.9])	625 (17.6%, [16.3-18.8])	1,085
	A little	-	-	283 (19%, [17-21])	650 (22.9%, [21.4-24.5])	948 (26.6%, [25.2-28.1])	1,881
	Not at all	-	-	83 (5.6%, [4.5-6.8])	282 (9.9%, [8.9-11.1])	266 (7.5%, [6.7-8.4])	631
	Don't know / PNA / Non-response	-	-	429 (28.7%, [26.5-31.1])	749 (26.4%, [24.8-28.1])	862 (24.2%, [22.8-25.6])	4,623
	No symptoms reported	-	-	541 (36.2%, [33.8-38.7])	851 (30%, [28.4-31.7])	859 (24.1%, [22.8-25.6])	4,914
Days since onset of first symptom	Mean (SD)	7.08 (3.19)	7.58 (3.14)	6.85 (3.19)	6.62 (3.04)	6.06 (2.94)	7.06 (3.16)
Symptom in past 7 days	Loss or change of sense of smell	399 (13.4%, [12.3-14.7])	323 (14.2%, [12.8-15.7])	367 (24.6%, [22.5-26.8])	214 (7.5%, [6.6-8.6])	349 (9.8%, [8.9-10.8])	1,652
	Loss or change of sense of taste	355 (11.9%, [10.8-13.2])	329 (14.5%, [13.1-16])	340 (22.8%, [20.7-25])	258 (9.1%, [8.1-10.2])	408 (11.5%, [10.5-12.5])	1,690
	New persistent	260 (8.8%, [7.8-9.8])	326 (14.3%, [12.9-15.8])	380 (25.5%, [23.3-27.7])	703 (24.8%, [23.2-26.4])	1,249 (35.1%, [33.5-36.7])	2,918

	cough						
	Fever	292 (9.8%, [8.8-11])	262 (11.5%, [10.3-12.9])	305 (20.4%, [18.5-22.5])	498 (17.6%, [16.2-19])	922 (25.9%, [24.5-27.4])	2,279
	Runny nose	389 (13.1%, [11.9-14.4])	384 (16.9%, [15.4-18.5])	505 (33.8%, [31.5-36.3])	1,146 (40.4%, [38.6-42.2])	1,825 (51.3%, [49.6-52.9])	4,249
	Sneezing	411 (13.8%, [12.6-15.1])	404 (17.8%, [16.2-19.4])	436 (29.2%, [27-31.6])	1,017 (35.9%, [34.1-37.7])	1,572 (44.2%, [42.5-45.8])	3,840
	Blocked nose	387 (13%, [11.9-14.3])	401 (17.6%, [16.1-19.2])	402 (26.9%, [24.7-29.2])	804 (28.4%, [26.7-30])	1,383 (38.8%, [37.3-40.5])	3,377
	Sore eyes	240 (8.1%, [7.2-9.1])	215 (9.5%, [8.3-10.7])	199 (13.3%, [11.7-15.1])	358 (12.6%, [11.5-13.9])	568 (16%, [14.8-17.2])	1,580
	Sore throat	409 (13.8%, [12.6-15.1])	386 (17%, [15.5-18.6])	374 (25.1%, [22.9-27.3])	1,073 (37.8%, [36.1-39.6])	1,734 (48.7%, [47.1-50.4])	3,976
	Hoarse voice	194 (6.5%, [5.7-7.5])	184 (8.1%, [7-9.3])	221 (14.8%, [13.1-16.7])	631 (22.3%, [20.8-23.8])	1,043 (29.3%, [27.8-30.8])	2,273
	Headache	740 (24.9%, [23.4-26.5])	670 (29.5%, [27.6-31.4])	553 (37%, [34.6-39.5])	1,130 (39.9%, [38.1-41.7])	1,684 (47.3%, [45.7-48.9])	4,777
	Dizziness	237 (8%, [7.1-9])	223 (9.8%, [8.6-11.1])	187 (12.5%, [10.9-14.3])	309 (10.9%, [9.8-12.1])	536 (15.1%, [13.9-16.3])	1,492
	Appetite loss	308 (10.4%, [9.3-11.5])	319 (14%, [12.7-15.5])	238 (15.9%, [14.2-17.9])	292 (10.3%, [9.2-11.5])	562 (15.8%, [14.6-17])	1,719
	Nausea/vomiting	153 (5.1%, [4.4-6])	141 (6.2%, [5.3-7.3])	107 (7.2%, [6-8.6])	185 (6.5%, [5.7-7.5])	289 (8.1%, [7.3-9.1])	875
	Diarrhoea	180 (6.1%, [5.3-7])	171 (7.5%, [6.5-8.7])	122 (8.2%, [6.9-9.7])	209 (7.4%, [6.5-8.4])	280 (7.9%, [7-8.8])	962
	Abdominal pain / belly ache	180 (6.1%, [5.3-7])	165 (7.3%, [6.3-8.4])	103 (6.9%, [5.7-8.3])	192 (6.8%, [5.9-7.8])	328 (9.2%, [8.3-10.2])	968
	Shortness of breath	223 (7.5%, [6.6-8.5])	232 (10.2%, [9-11.5])	185 (12.4%, [10.8-14.2])	353 (12.5%, [11.3-13.7])	575 (16.2%, [15-17.4])	1,568
	Tight chest	204 (6.9%, [6-7.8])	203 (8.9%, [7.8-10.2])	139 (9.3%, [7.9-10.9])	270 (9.5%, [8.5-10.7])	423 (11.9%, [10.9-13])	1,239
	Chest pain	83 (2.8%, [2.3-3.4])	87 (3.8%, [3.1-4.7])	50 (3.3%, [2.5-4.4])	119 (4.2%, [3.5-5])	202 (5.7%, [5-6.5])	541
	Chills	309 (10.4%, [9.4-11.5])	330 (14.5%, [13.1-16])	252 (16.9%, [15.1-18.9])	495 (17.5%, [16.1-18.9])	880 (24.7%, [23.3-26.2])	2,266
	Difficulty sleeping	349 (11.7%, [10.6-13])	323 (14.2%, [12.8-15.7])	263 (17.6%, [15.8-19.6])	520 (18.3%, [17-19.8])	788 (22.1%, [20.8-23.5])	2,243
	Tiredness	727 (24.5%, [23-26])	700 (30.8%, [28.9-32.7])	534 (35.8%, [33.4-38.2])	1,073 (37.8%, [36.1-39.6])	1,658 (46.6%, [44.9-48.2])	4,692
	Severe fatigue	149 (5%, [4.3-5.9])	167 (7.3%, [6.3-8.5])	118 (7.9%, [6.6-9.4])	168 (5.9%, [5.1-6.9])	331 (9.3%, [8.4-10.3])	933
	Numbness/tingling	110 (3.7%, [3.1-4.4])	90 (4%, [3.2-4.8])	58 (3.9%, [3-5])	126 (4.4%, [3.7-5.3])	214 (6%, [5.3-6.8])	598
	Heavy arms/legs	196 (6.6%, [5.8-7.5])	193 (8.5%, [7.4-9.7])	100 (6.7%, [5.5-8.1])	228 (8%, [7.1-9.1])	419 (11.8%, [10.8-12.9])	1,136
	Muscle aches	549 (18.5%, [17.1-19.9])	463 (20.4%, [18.7-22.1])	372 (24.9%, [22.8-27.2])	766 (27%, [25.4-28.7])	1,198 (33.7%, [32.1-35.2])	3,348
First reported symptoms	Loss or change of sense of smell	109 (3.7%, [3.1-4.4])	82 (3.6%, [2.9-4.5])	73 (4.9%, [3.9-6.1])	36 (1.3%, [0.9-1.8])	46 (1.3%, [1-1.7])	346
	Loss or change of sense of taste	96 (3.2%, [2.7-3.9])	83 (3.6%, [3-4.5])	66 (4.4%, [3.5-5.6])	38 (1.3%, [1-1.8])	59 (1.7%, [1.3-2.1])	342
	New persistent	125 (4.2%, [3.5-5])	148 (6.5%, [5.6-7.6])	168 (11.3%, [9.7-13])	255 (9%, [8-10.1])	375 (10.5%, [9.6-11.6])	1,071

cough						
Fever	112 (3.8%, [3.1-4.5])	104 (4.6%, [3.8-5.5])	83 (5.6%, [4.5-6.8])	133 (4.7%, [4-5.5])	232 (6.5%, [5.8-7.4])	664
Runny nose	129 (4.3%, [3.7-5.1])	110 (4.8%, [4-5.8])	176 (11.8%, [10.2-13.5])	363 (12.8%, [11.6-14.1])	561 (15.8%, [14.6-17])	1,339
Sneezing	125 (4.2%, [3.5-5])	122 (5.4%, [4.5-6.4])	146 (9.8%, [8.4-11.4])	304 (10.7%, [9.6-11.9])	442 (12.4%, [11.4-13.5])	1,139
Blocked nose	120 (4%, [3.4-4.8])	138 (6.1%, [5.2-7.1])	98 (6.6%, [5.4-7.9])	203 (7.2%, [6.3-8.2])	304 (8.5%, [7.7-9.5])	863
Sore eyes	77 (2.6%, [2.1-3.2])	65 (2.9%, [2.2-3.6])	42 (2.8%, [2.1-3.8])	91 (3.2%, [2.6-3.9])	147 (4.1%, [3.5-4.8])	422
Sore throat	206 (6.9%, [6.1-7.9])	183 (8%, [7-9.2])	186 (12.5%, [10.9-14.2])	620 (21.9%, [20.4-23.4])	1,084 (30.4%, [29-32])	2,279
Hoarse voice	50 (1.7%, [1.3-2.2])	38 (1.7%, [1.2-2.3])	59 (4%, [3.1-5.1])	159 (5.6%, [4.8-6.5])	212 (6%, [5.2-6.8])	518
Headache	384 (12.9%, [11.8-14.2])	340 (14.9%, [13.5-16.5])	250 (16.7%, [14.9-18.7])	508 (17.9%, [16.6-19.4])	716 (20.1%, [18.8-21.5])	2,198
Dizziness	76 (2.6%, [2-3.2])	65 (2.9%, [2.2-3.6])	41 (2.7%, [2-3.7])	64 (2.3%, [1.8-2.9])	116 (3.3%, [2.7-3.9])	362
Appetite loss	69 (2.3%, [1.8-2.9])	78 (3.4%, [2.8-4.3])	48 (3.2%, [2.4-4.2])	49 (1.7%, [1.3-2.3])	85 (2.4%, [1.9-2.9])	329
Nausea/vomiting	38 (1.3%, [0.9-1.8])	30 (1.3%, [0.9-1.9])	28 (1.9%, [1.3-2.7])	44 (1.6%, [1.2-2.1])	81 (2.3%, [1.8-2.8])	221
Diarrhoea	47 (1.6%, [1.2-2.1])	46 (2%, [1.5-2.7])	30 (2%, [1.4-2.9])	46 (1.6%, [1.2-2.2])	69 (1.9%, [1.5-2.4])	238
Abdominal pain / belly ache	58 (2%, [1.5-2.5])	44 (1.9%, [1.4-2.6])	18 (1.2%, [0.8-1.9])	43 (1.5%, [1.1-2])	70 (2%, [1.6-2.5])	233
Shortness of breath	69 (2.3%, [1.8-2.9])	67 (2.9%, [2.3-3.7])	32 (2.1%, [1.5-3])	77 (2.7%, [2.2-3.4])	109 (3.1%, [2.5-3.7])	354
Tight chest	45 (1.5%, [1.1-2])	48 (2.1%, [1.6-2.8])	31 (2.1%, [1.5-2.9])	51 (1.8%, [1.4-2.4])	77 (2.2%, [1.7-2.7])	252
Chest pain	19 (0.6%, [0.4-1])	11 (0.5%, [0.3-0.9])	10 (0.7%, [0.4-1.2])	20 (0.7%, [0.5-1.1])	36 (1%, [0.7-1.4])	96
Chills	103 (3.5%, [2.9-4.2])	131 (5.8%, [4.9-6.8])	60 (4%, [3.1-5.1])	139 (4.9%, [4.2-5.8])	271 (7.6%, [6.8-8.5])	704
Difficulty sleeping	114 (3.8%, [3.2-4.6])	104 (4.6%, [3.8-5.5])	77 (5.2%, [4.1-6.4])	118 (4.2%, [3.5-5])	184 (5.2%, [4.5-5.9])	597
Tiredness	314 (10.6%, [9.5-11.7])	264 (11.6%, [10.4-13])	202 (13.5%, [11.9-15.4])	385 (13.6%, [12.4-14.9])	602 (16.9%, [15.7-18.2])	1,767
Severe fatigue	57 (1.9%, [1.5-2.5])	49 (2.2%, [1.6-2.8])	35 (2.3%, [1.7-3.2])	48 (1.7%, [1.3-2.2])	93 (2.6%, [2.1-3.2])	282
Numbness/tingling	30 (1%, [0.7-1.4])	25 (1.1%, [0.7-1.6])	12 (0.8%, [0.5-1.4])	31 (1.1%, [0.8-1.5])	37 (1%, [0.8-1.4])	135
Heavy arms/legs	58 (2%, [1.5-2.5])	53 (2.3%, [1.8-3])	21 (1.4%, [0.9-2.1])	44 (1.6%, [1.2-2.1])	86 (2.4%, [2-3])	262
Muscle aches	249 (8.4%, [7.4-9.4])	197 (8.7%, [7.6-9.9])	121 (8.1%, [6.8-9.6])	261 (9.2%, [8.2-10.3])	378 (10.6%, [9.6-11.7])	1,206

Supplementary Table 3 Odds ratios for swab positivity based on presence or absence of any of 26 symptoms surveyed in N=1,542,510 participants across selected rounds of REACT-1. ORs are derived from logistic regression models with swab positive (1/0) as the outcome variable, adjusted for age group, sex and vaccination status. 95% confidence intervals are shown.

**Symptom**	**Wild-type Rounds 2-7**	**Alpha Rounds 8-10**	**Delta Rounds 13–15**	**BA.1 Rounds 17–19**	**BA.2 Rounds 17–19**
	June – Dec 2020	*Jan – Mar 2021*	*June – Nov 2021*	*Jan–Mar 2022*	*Jan–Mar 2022*
**Any of 26 symptoms**	5.16 (4.79,5.55)	6.01 (5.12,7.06)	9.53 (8.55,10.6)	9.61 (8.82,10.5)	12.9 (11.9,14.0)
Loss or change of sense of smell	49.7 (44.3,55.7)	37.8 (28.6,50.0)	73.4 (64.2,83.9)	12.9 (11.1,15.1)	17.2 (15.1,19.5)
Loss or change of sense of taste	35.9 (31.9,40.4)	38.9 (29.9,50.6)	68.1 (59.4,78.0)	16.0 (13.9,18.5)	21.3 (18.9,24.0)
New persistent cough	13.9 (12.2,15.8)	31.9 (24.9,40.9)	25.9 (22.9,29.3)	16.4 (14.9,18.0)	26.2 (24.2,28.3)
Fever	13.3 (11.7,15.0)	16.2 (12.2,21.4)	26.3 (23.0,30.1)	18.4 (16.5,20.5)	30.2 (27.7,33.0)
Appetite loss	12.0 (10.6,13.6)	15.2 (11.8,19.7)	17.8 (15.4,20.7)	8.56 (7.50,9.77)	13.6 (12.3,15.1)
Chills	11.5 (10.2,13.0)	9.79 (7.62,12.6)	19.3 (16.7,22.2)	11.5 (10.4,12.8)	17.9 (16.4,19.4)
Severe fatigue	7.70 (6.51,9.11)	8.74 (6.28,12.1)	10.5 (8.69,12.7)	5.30 (4.49,6.26)	8.16 (7.21,9.25)
Muscle aches	7.32 (6.66,8.04)	7.44 (6.06,9.15)	10.3 (9.16,11.7)	9.02 (8.25,9.87)	12.5 (11.6,13.5)
Hoarse voice	5.88 (5.07,6.81)	8.80 (6.40,12.1)	9.28 (8.00,10.8)	12.8 (11.7,14.2)	18.9 (17.4,20.5)
Heavy arms/legs	5.87 (5.07,6.80)	6.96 (5.06,9.56)	6.19 (5.04,7.61)	6.07 (5.25,7.02)	9.47 (8.46,10.6)
Tight chest	5.57 (4.82,6.44)	6.13 (4.45,8.45)	7.91 (6.59,9.51)	6.40 (5.59,7.31)	7.96 (7.12,8.90)
Headache	5.49 (5.04,5.98)	5.88 (4.90,7.07)	9.83 (8.80,11.0)	8.28 (7.62,8.98)	11.2 (10.5,12.1)
Blocked nose	5.45 (4.89,6.08)	8.26 (6.63,10.3)	11.1 (9.85,12.5)	8.47 (7.75,9.25)	13.2 (12.3,14.3)
Tiredness	5.33 (4.89,5.80)	6.75 (5.64,8.07)	8.27 (7.41,9.23)	6.78 (6.25,7.36)	9.76 (9.09,10.5)
Chest pain	4.59 (3.68,5.72)	3.75 (2.24,6.28)	4.93 (3.67,6.62)	4.86 (3.99,5.91)	6.40 (5.48,7.47)
Dizziness	4.58 (4.00,5.24)	5.46 (4.10,7.28)	7.65 (6.53,8.96)	5.46 (4.82,6.19)	7.76 (7.02,8.57)
Shortness of breath	4.45 (3.88,5.11)	6.39 (4.90,8.34)	7.40 (6.31,8.66)	5.16 (4.58,5.80)	7.02 (6.38,7.73)
Nausea/vomiting	4.34 (3.68,5.12)	5.95 (4.26,8.31)	6.40 (5.23,7.84)	5.05 (4.32,5.91)	5.90 (5.18,6.72)
Sneezing	4.28 (3.85,4.76)	7.06 (5.70,8.76)	10.0 (8.92,11.3)	10.9 (10.0,11.8)	15.0 (14.0,16.1)
Sore eyes	4.22 (3.69,4.82)	5.32 (4.03,7.02)	8.15 (7.00,9.49)	5.63 (5.00,6.34)	7.53 (6.83,8.29)
Runny nose	4.04 (3.62,4.50)	5.36 (4.28,6.72)	10.8 (9.67,12.1)	9.81 (9.05,10.6)	14.9 (13.9,16.0)
Diarrhoea	3.84 (3.30,4.48)	4.96 (3.57,6.88)	5.62 (4.64,6.81)	4.44 (3.82,5.15)	4.52 (3.96,5.16)
Sore throat	3.76 (3.38,4.19)	6.56 (5.24,8.20)	7.22 (6.38,8.18)	11.6 (10.7,12.6)	18.0 (16.8,19.4)
Difficulty sleeping	3.30 (2.95,3.70)	3.23 (2.51,4.15)	5.39 (4.70,6.18)	4.48 (4.05,4.95)	5.45 (5.01,5.94)
Abdominal pain / belly ache	2.89 (2.48,3.37)	2.37 (1.62,3.47)	3.74 (3.06,4.58)	3.13 (2.68,3.65)	4.17 (3.70,4.71)
Numbness/tingling	2.80 (2.31,3.39)	2.66 (1.72,4.12)	2.95 (2.27,3.84)	2.78 (2.30,3.35)	4.00 (3.46,4.63)

Supplementary Table 4 Unadjusted odds ratios for swab positivity based on presence or absence of any of 26 symptoms surveyed in N=1,542,510 participants across selected rounds of REACT-1. ORs are derived from logistic regression models with swab positive (1/0) as the outcome variable. 95% confidence intervals are shown.

**Symptom**	**Wild-type Rounds 2-7**	**Alpha Rounds 8-10**	**Delta Rounds 13–15**	**BA.1 Rounds 17–19**	**BA.2 Rounds 17–19**
	June – Dec 2020	*Jan – Mar 2021*	*June – Nov 2021*	*Jan–Mar 2022*	*Jan–Mar 2022*
**Any of 26 symptoms**	5.01 (4.68,5.36)	6.50 (6.02,7.02)	8.25 (7.52,9.06)	8.23 (7.64,8.86)	10.5 (9.76,11.2)
Loss or change of sense of smell	53.6 (47.9,60.0)	52.1 (45.7,59.4)	75.5 (66.6,85.7)	13.7 (11.8,15.9)	18.0 (16.0,20.4)
Loss or change of sense of taste	38.3 (34.1,43.0)	46.5 (40.9,52.9)	69.0 (60.6,78.5)	17.0 (14.8,19.5)	21.7 (19.4,24.4)
New persistent cough	15.4 (13.5,17.5)	31.3 (27.7,35.5)	26.7 (23.7,30.0)	16.5 (15.1,18.1)	26.7 (24.8,28.7)
Fever	14.4 (12.8,16.3)	19.5 (17.1,22.3)	28.0 (24.7,31.8)	18.9 (17.0,21.0)	30.9 (28.4,33.6)
Runny nose	4.37 (3.92,4.86)	5.70 (5.11,6.36)	10.9 (9.79,12.0)	9.56 (8.87,10.3)	14.2 (13.3,15.2)
Sneezing	4.60 (4.15,5.11)	6.71 (6.03,7.47)	10.1 (9.10,11.3)	10.7 (9.89,11.5)	14.6 (13.7,15.7)
Blocked nose	6.00 (5.39,6.67)	8.68 (7.78,9.67)	11.1 (9.93,12.4)	8.48 (7.80,9.21)	13.3 (12.4,14.2)
Sore eyes	4.40 (3.86,5.02)	5.33 (4.63,6.14)	8.24 (7.12,9.55)	5.80 (5.18,6.50)	7.56 (6.89,8.30)
Sore throat	4.09 (3.68,4.54)	6.78 (6.08,7.56)	7.68 (6.85,8.61)	11.2 (10.4,12.1)	17.0 (15.9,18.1)
Hoarse voice	6.13 (5.29,7.10)	9.60 (8.23,11.2)	9.61 (8.33,11.1)	13.3 (12.1,14.6)	18.9 (17.5,20.4)
Headache	5.70 (5.25,6.18)	6.40 (5.86,7.00)	9.52 (8.61,10.5)	7.94 (7.37,8.57)	10.5 (9.87,11.2)
Dizziness	4.86 (4.25,5.55)	6.18 (5.37,7.10)	7.89 (6.78,9.18)	5.57 (4.93,6.28)	7.93 (7.21,8.72)
Appetite loss	12.9 (11.4,14.5)	18.2 (16.1,20.6)	18.3 (15.9,21.0)	8.63 (7.61,9.80)	13.9 (12.7,15.3)
Nausea/vomiting	4.83 (4.10,5.69)	5.99 (5.04,7.12)	6.85 (5.65,8.32)	5.01 (4.30,5.84)	6.27 (5.53,7.10)
Diarrhoea	4.20 (3.61,4.88)	5.94 (5.07,6.95)	6.13 (5.10,7.37)	4.64 (4.02,5.36)	4.93 (4.34,5.58)
Abdominal pain / belly ache	3.14 (2.70,3.66)	3.89 (3.31,4.56)	3.93 (3.23,4.78)	3.20 (2.76,3.71)	4.41 (3.93,4.96)
Shortness of breath	4.53 (3.95,5.19)	5.94 (5.18,6.81)	7.52 (6.46,8.76)	5.26 (4.70,5.90)	7.02 (6.41,7.70)
Tight chest	5.98 (5.19,6.90)	7.67 (6.62,8.88)	8.74 (7.34,10.4)	6.66 (5.85,7.58)	8.41 (7.56,9.35)
Chest pain	5.08 (4.08,6.33)	6.21 (4.99,7.72)	5.50 (4.15,7.29)	5.10 (4.22,6.16)	6.94 (5.99,8.05)
Chills	12.2 (10.8,13.7)	11.1 (9.85,12.5)	20.3 (17.7,23.2)	11.8 (10.7,13.1)	18.1 (16.7,19.6)
Difficulty sleeping	3.51 (3.14,3.93)	3.94 (3.50,4.43)	5.74 (5.04,6.54)	4.56 (4.14,5.02)	5.65 (5.22,6.13)
Tiredness	5.48 (5.05,5.95)	7.21 (6.60,7.87)	8.34 (7.54,9.23)	6.72 (6.23,7.25)	9.38 (8.79,10.0)
Severe fatigue	8.37 (7.08,9.89)	10.6 (8.99,12.4)	11.1 (9.26,13.4)	5.45 (4.64,6.39)	8.70 (7.73,9.80)
Numbness/tingling	2.85 (2.36,3.45)	3.02 (2.44,3.74)	3.03 (2.35,3.92)	2.82 (2.35,3.39)	3.88 (3.37,4.47)
Heavy arms/legs	6.04 (5.22,6.99)	8.20 (7.06,9.52)	6.56 (5.38,8.00)	6.42 (5.58,7.38)	9.68 (8.70,10.8)
Muscle aches	7.49 (6.83,8.21)	7.82 (7.06,8.66)	10.5 (9.32,11.7)	9.02 (8.28,9.81)	12.1 (11.3,13.0)

Supplementary Table 5 Results from log-linear regression of symptom count as a function of BA.1 / BA.1 infection, age group, sex, booster vaccine received (y/n), weeks since most recent vaccine, prior COVID-19 (28 days or more before testing), weeks since symptom onset, and calendar time (since 1 Jan 2021) among 5,637 double- or triple-vaccinated swab-positive individuals with either BA.2 or BA.1 lineage infection. Errors are modelled as Poisson distributed. Odd Ratios and 95% confidence intervals are shown. Each column shows the addition of one covariate to the model. BA.2 infection (vs BA.1) is associated with reporting of more symptoms. This effect is robust to adjustment.

Variable	Category	Crude model	Plus age	Plus sex	Plus boosted	Plus weeks since vaccination	Plus prior COVID-19	Plus weeks since symptom onset	Plus calendar time (weeks)
Omicron variant	BA.1 [ref]	-	-	-	-	-	-	-	-
	**BA.2**	**1.3 (1.27,1.33) ******	**1.29 (1.26,1.32) ******	**1.29 (1.26,1.32) ******	**1.29 (1.26,1.32) ******	**1.28 (1.25,1.31) ******	**1.29 (1.25,1.32) ******	**1.19 (1.16,1.22) ******	**1.14 (1.1,1.19) ******
Age	18-24 [ref]		-	-	-	-	-	-	-
	25-34		1.06 (1,1.12) *	1.07 (1.02,1.13) **	1.07 (1.02,1.13) *	1.07 (1.01,1.13) *	1.08 (1.03,1.14) **	1.02 (0.96,1.07)	1.02 (0.97,1.08)
	35-44		1.09 (1.04,1.15) ***	1.11 (1.05,1.17) ***	1.11 (1.05,1.17) ***	1.1 (1.05,1.16) ***	1.11 (1.05,1.17) ***	1.06 (1,1.12) *	1.06 (1.01,1.12) *
	45-54		1.12 (1.06,1.18) ****	1.15 (1.09,1.21) ****	1.14 (1.08,1.2) ****	1.14 (1.08,1.2) ****	1.13 (1.07,1.19) ****	1.07 (1.02,1.13) *	1.08 (1.02,1.14) **
	55-64		1.01 (0.95,1.06)	1.04 (0.99,1.1)	1.03 (0.98,1.09)	1.03 (0.98,1.09)	1.03 (0.97,1.09)	1 (0.95,1.06)	1.01 (0.96,1.07)
	65-74		0.83 (0.79,0.88) ****	0.87 (0.82,0.92) ****	0.87 (0.82,0.92) ****	0.86 (0.81,0.91) ****	0.85 (0.8,0.9) ****	0.85 (0.8,0.9) ****	0.86 (0.81,0.91) ****
Sex	Female [ref]			0.73 (0.68,0.78) ****	0.72 (0.67,0.78) ****	0.72 (0.66,0.77) ****	0.7 (0.65,0.76) ****	0.77 (0.72,0.84) ****	0.78 (0.72,0.84) ****
	Male			-	-	-	-	-	-
Boosted (Yes)	No [ref]				0.79 (0.77,0.81) ****	0.79 (0.77,0.81) ****	0.79 (0.77,0.81) ****	0.85 (0.83,0.87) ****	0.85 (0.83,0.87) ****
	Yes				-	-	-	-	-
Weeks since last vaccination						1.04 (1,1.09)	1.02 (0.98,1.07)	0.95 (0.91,0.99) *	0.93 (0.89,0.98) **
Prior COVID-19 (28+ days ago)	No [ref]						1 (1,1)	1 (1,1)	1 (1,1)
	Yes						-	-	-
Weeks since symptom onset								0.86 (0.82,0.91) ****	0.86 (0.82,0.91) ****
Calendar time (weeks since 1 Jan)									1.04 (1.01,1.06) *

* p<0.05, ** p<0.01, *** p<0.001, *** p<0.0001

Supplementary Figure 1 Comparison of Ct values in N=6,395 swab-positive respondents with Omicron BA.2 and BA.1 variants in REACT-1 rounds 17–19, stratified by symptom status (presence of any of 26 surveyed symptoms). A two-tailed Wilcoxon signed rank test was used to compare the Ct value distributions in BA.2 vs BA.1, within symptomatics and asymptomatics. Ct values are lower in BA.2, in both asymptomatic and symptomatic respondents.

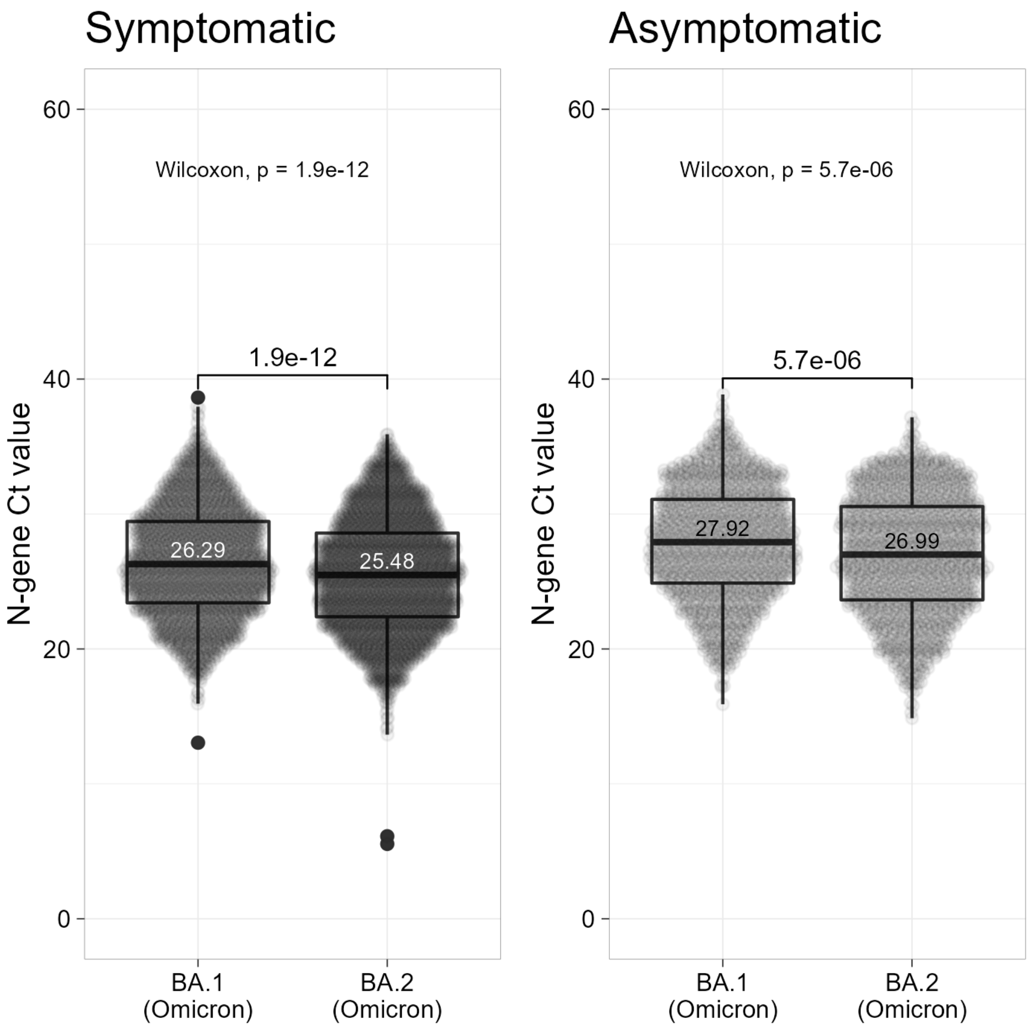

Supplementary Figure 2 Plot showing the odds ratios and 95% confidence intervals for symptoms in a pooled analysis of N=1,542,510 participants across five variant-phases. For each of the symptoms, a logistic model was fit with this specification:

y[PCR pos/neg] ~ *covariates* [age, sex, vaccination, calendar time] + *variant* [binary] + *symptom* [y/n] + *variant:symptom* [interaction]

For the reference category variant (Wild-type) we report the odds ratio on the symptom. For subsequent variants, we report the product of the symptom odds ratio and the interaction effect with the relevant variant. Error bars show 95% confidence intervals. The patterns of changes in odds ratios between variants are consistent with the patterns shown in the stratified logistic regression analysis in Figure 2.: ORs are higher for BA.2 than BA.1 for nearly all symptoms. Fever and cough have the highest ORs for BA.2 and BA.1, while loss or change of smell or taste have the highest ORs in all previous variants.

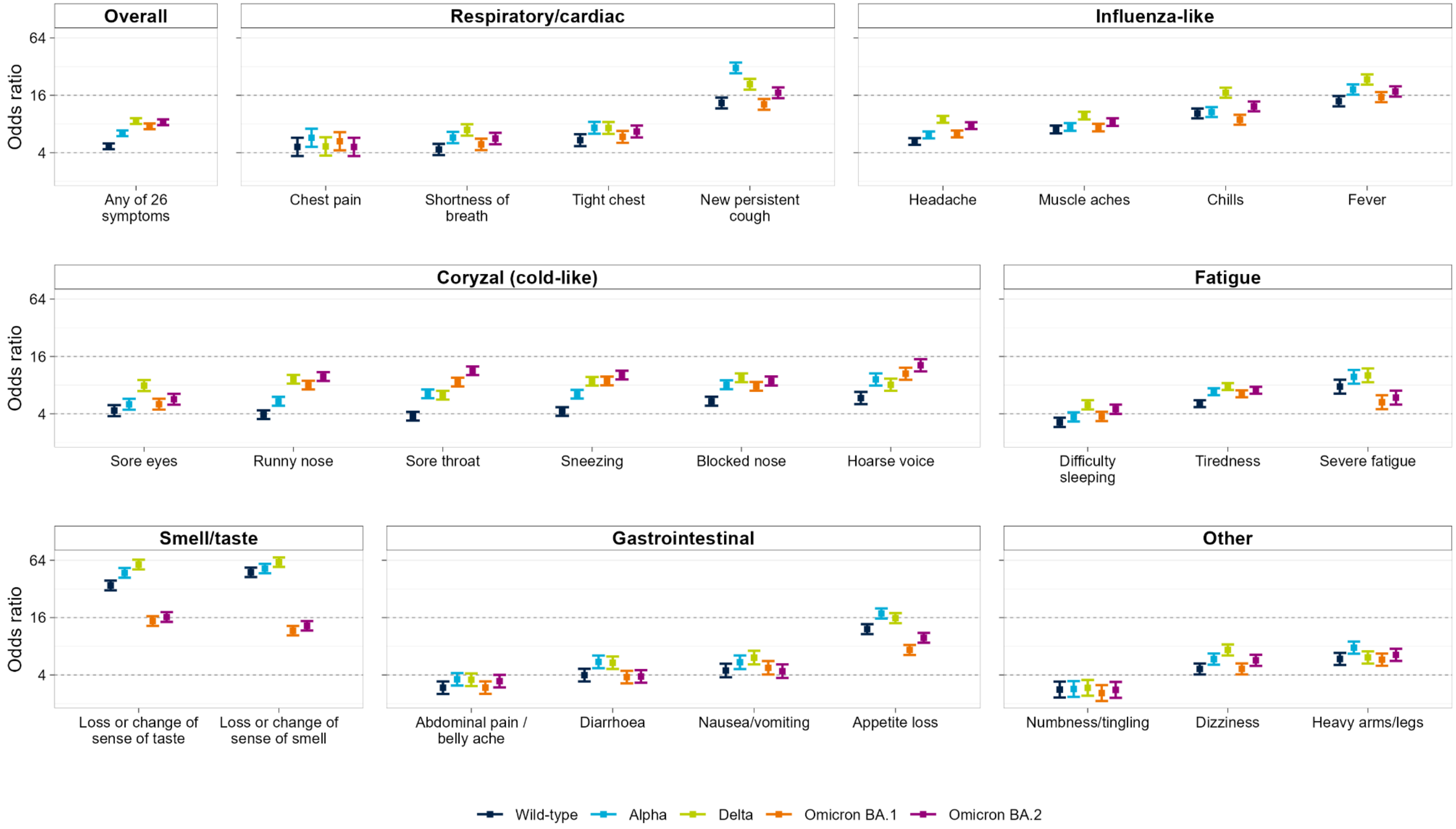

Supplementary Figure 3 Results of LASSO stability selection with swab positive/negative as the binary outcome variable and each of 26 symptoms as predictors, N=1,542,510 participants in five phases of REACT-1 corresponding to periods of dominance of five SARS-CoV-2 variants in England. Age, sex and, where appropriate, vaccination status are forced into the models as unpenalised variables; regression coefficients for the symptoms are constrained to be positive. The median, 5th and 95th percentiles of the Area Under the Curve (AUCs) are obtained from unpenalised models adding each symptom incrementally (from top to bottom) applied on un-seen holdout data.

A Wild type

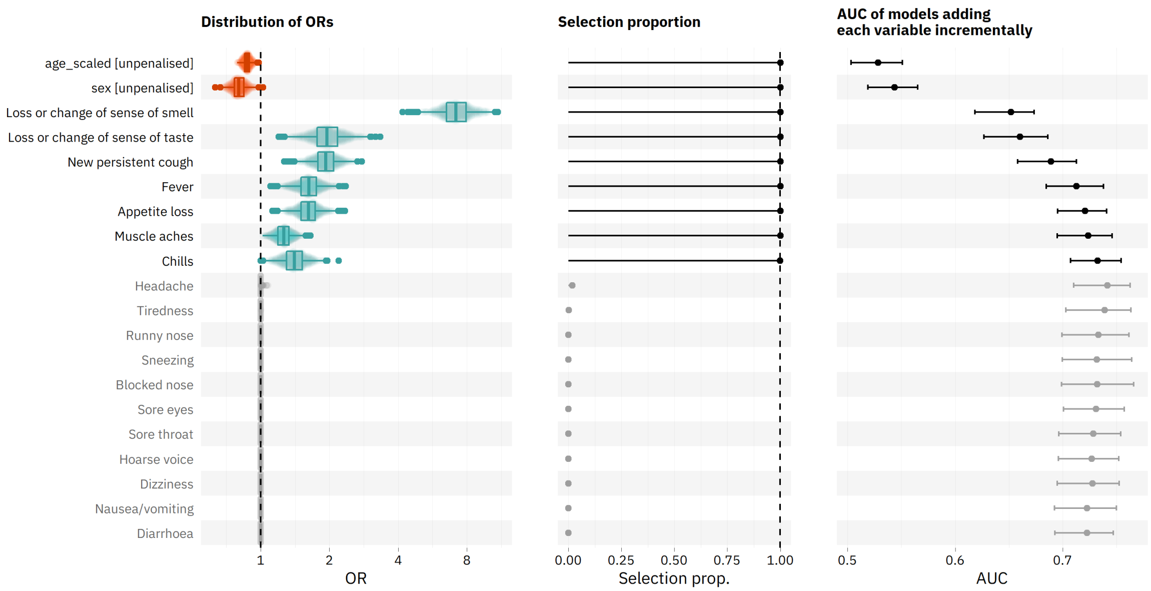

B Alpha

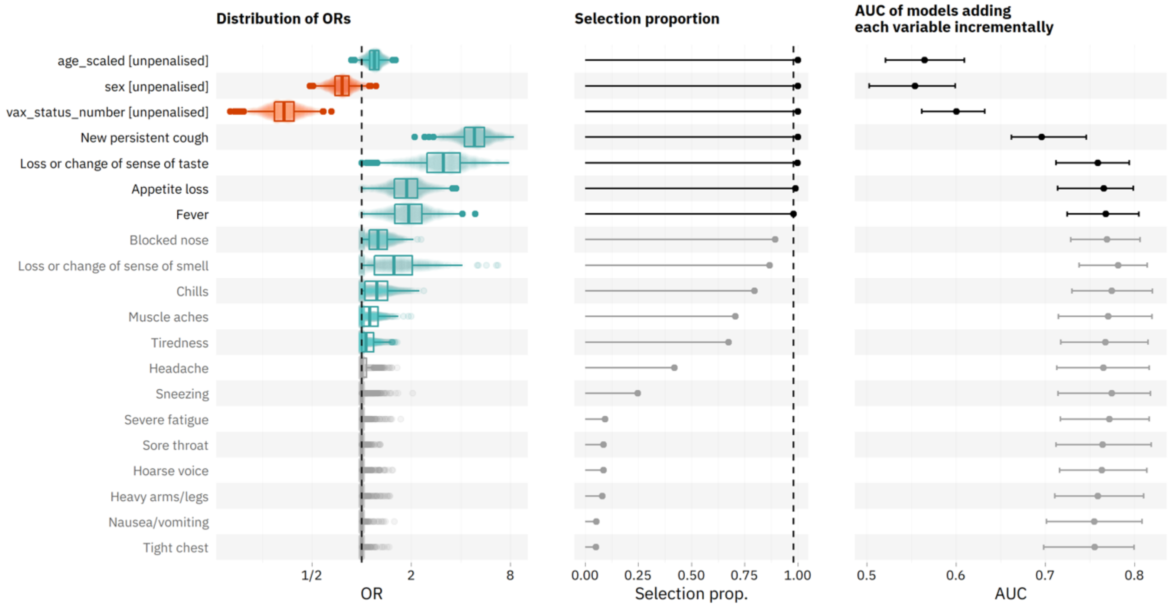

C Delta

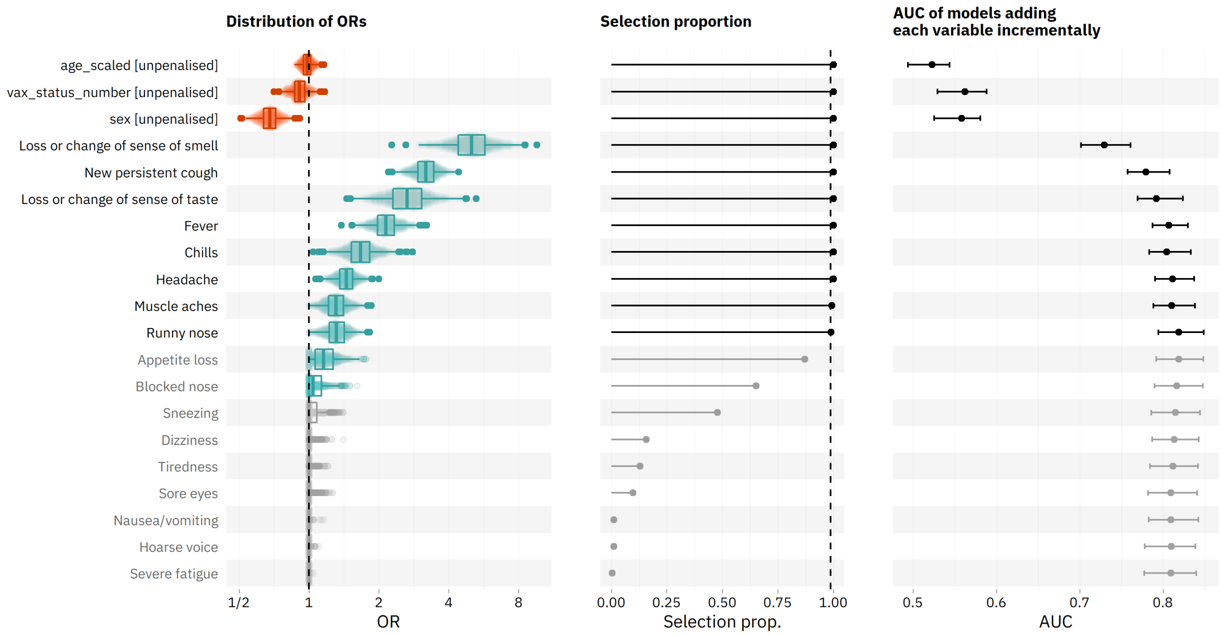

D Omicron BA.1

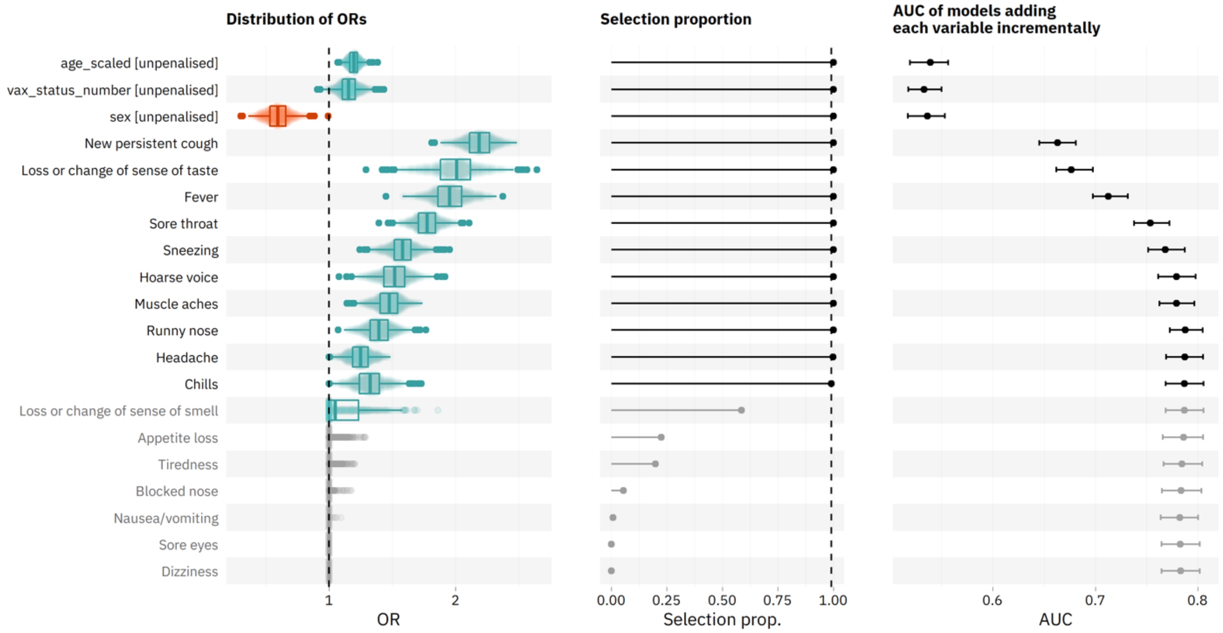

E Omicron BA.2

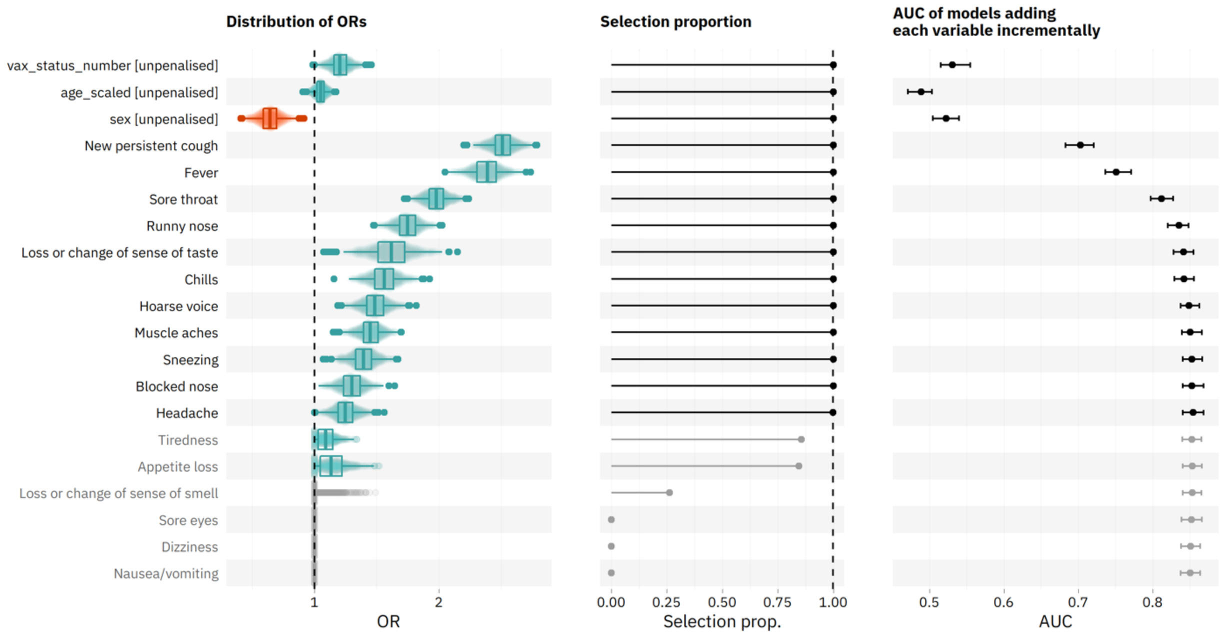

Supplementary Figure 4 Heatmaps showing co-occurrence of symptoms across variants among swab-positive individuals. Denominators for percentages are the number of swab positive individuals with each variant.

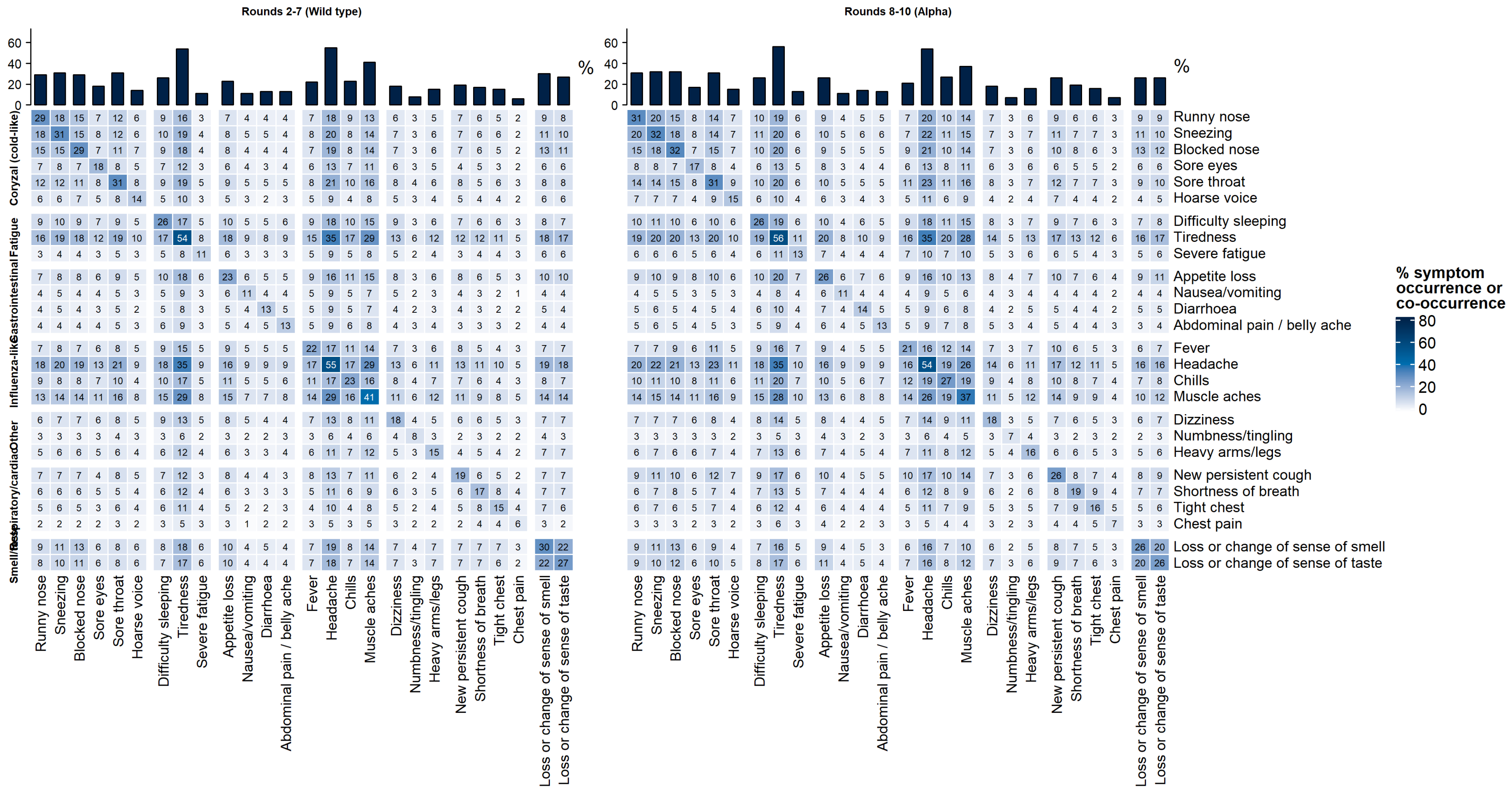

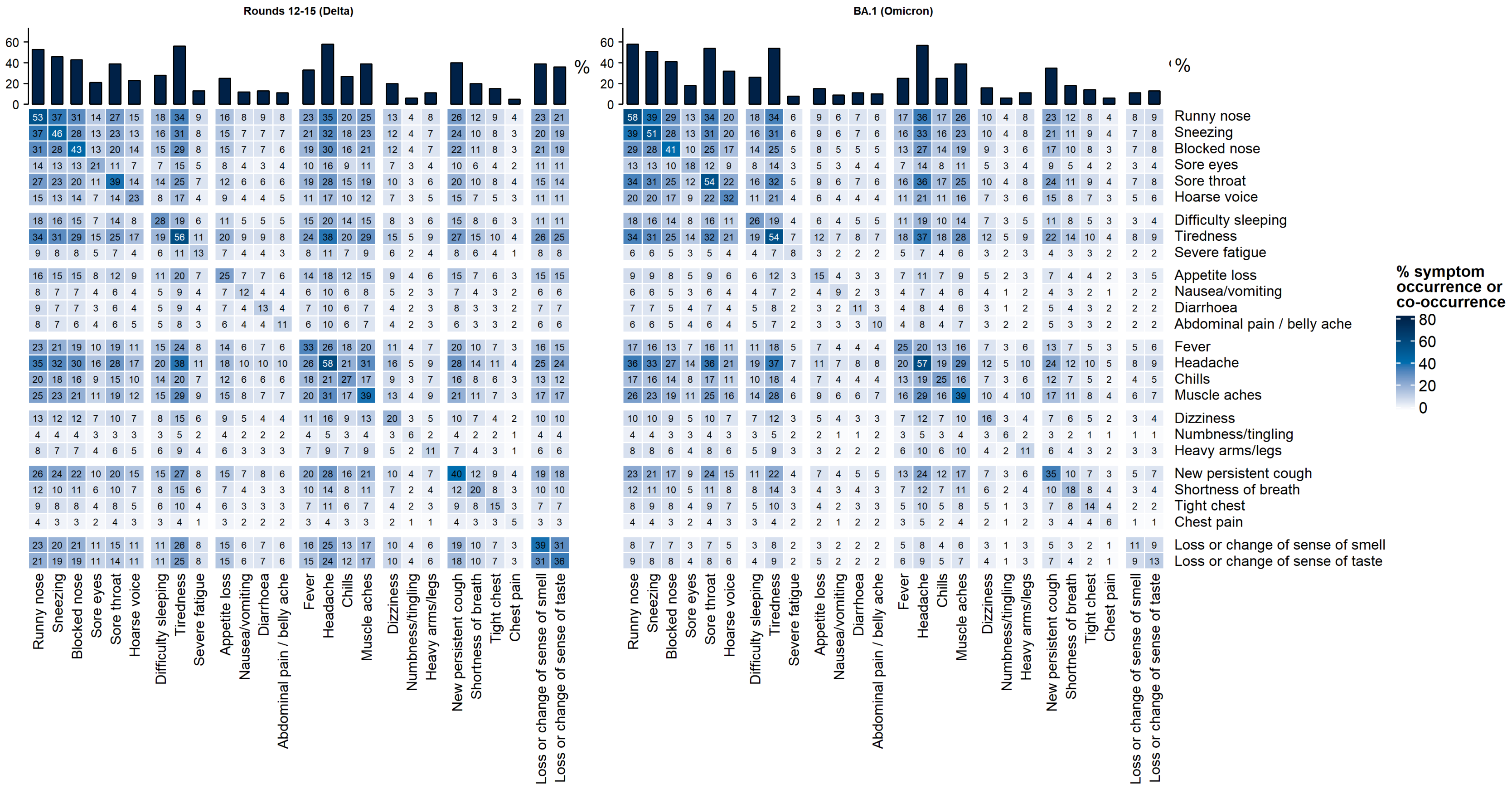

Rounds 12-15 (Delta)
BA.1 (Omicron)
%
% symptom occurrence or co-occurrence
Runny nose
Sneezing
Blocked nose
Sore eyes
Sore throat
Hoarse voice
Difficulty sleeping
Tiredness
Severe fatigue
Appetite loss
Nausea/vomiting
Diarrhoea
Abdominal pain / belly ache
Fever
Headache
Chills
Muscle aches
Dizziness
Numbness/tingling
Heavy arms/legs
New persistent cough
Shortness of breath
Tight chest
Chest pain
Loss or change of sense of smell
Loss or change of sense of taste

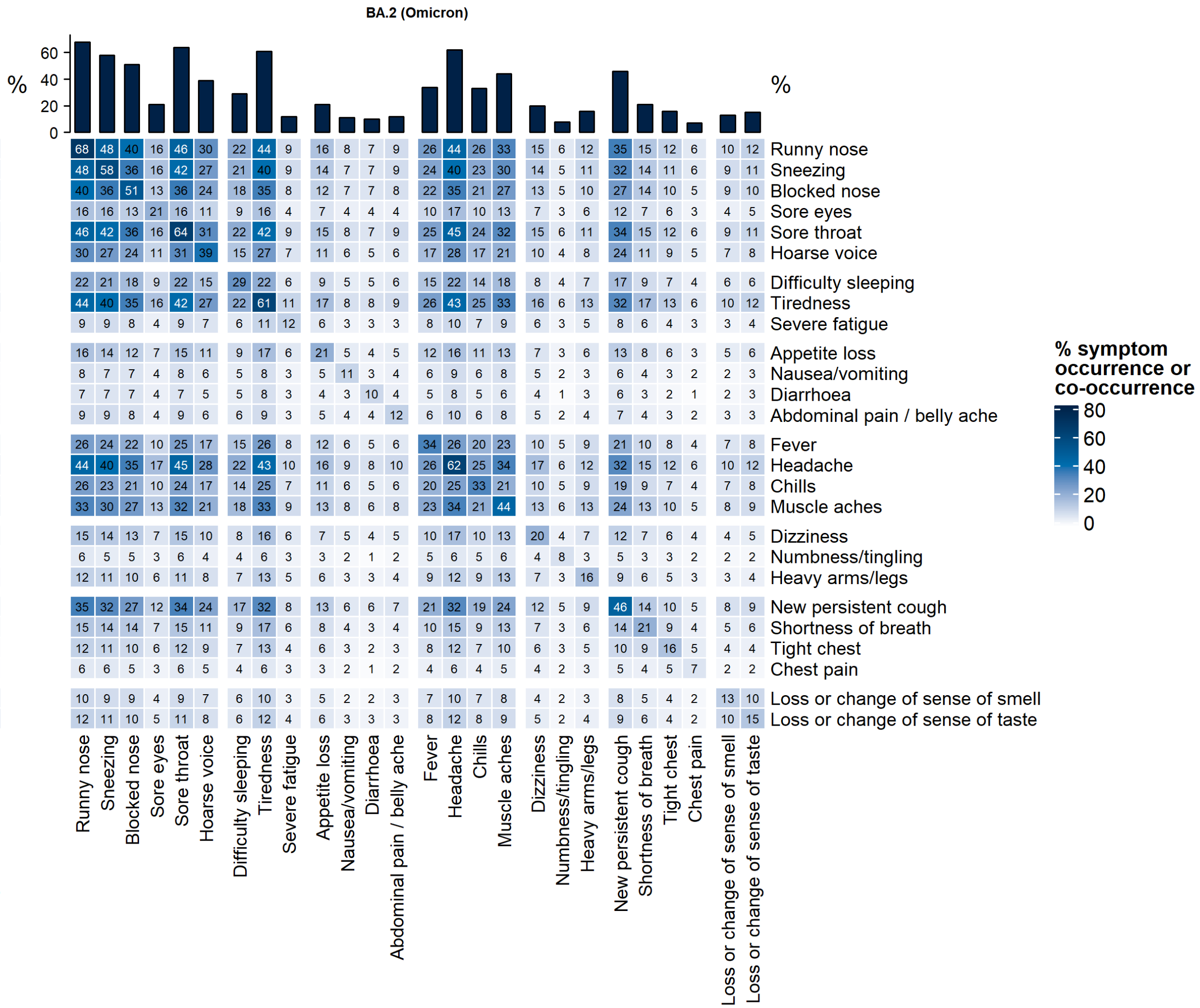
BA.2 (Omicron)
%
60
40
20
0
%
Runny nose
Sneezing
Blocked nose
Sore eyes
Sore throat
Hoarse voice
Difficulty sleeping
Tiredness
Severe fatigue
Appetite loss
Nausea/vomiting
Diarrhoea
Abdominal pain / belly ache
Fever
Headache
Chills
Muscle aches
Dizziness
Numbness/tingling
Heavy arms/legs
New persistent cough
Shortness of breath
Tight chest
Chest pain
Loss or change of sense of smell
Loss or change of sense of taste
% symptom occurrence or co-occurrence
80
60
40
20
0

Supplementary Figure 5 Heatmap showing co-occurrence of first symptoms among swab positive individuals in rounds 17–19 (5 January to 31 March 2022), when Omicron was dominant. The denominator for percentages is the number of swab positive individuals who reported experiencing one or more of 26 symptoms in the week prior to testing in rounds 17–19 (n=7,176). Sore throat, headache, and tiredness are the most common first symptoms and are the most commonly co-occurring pairs of first symptoms.

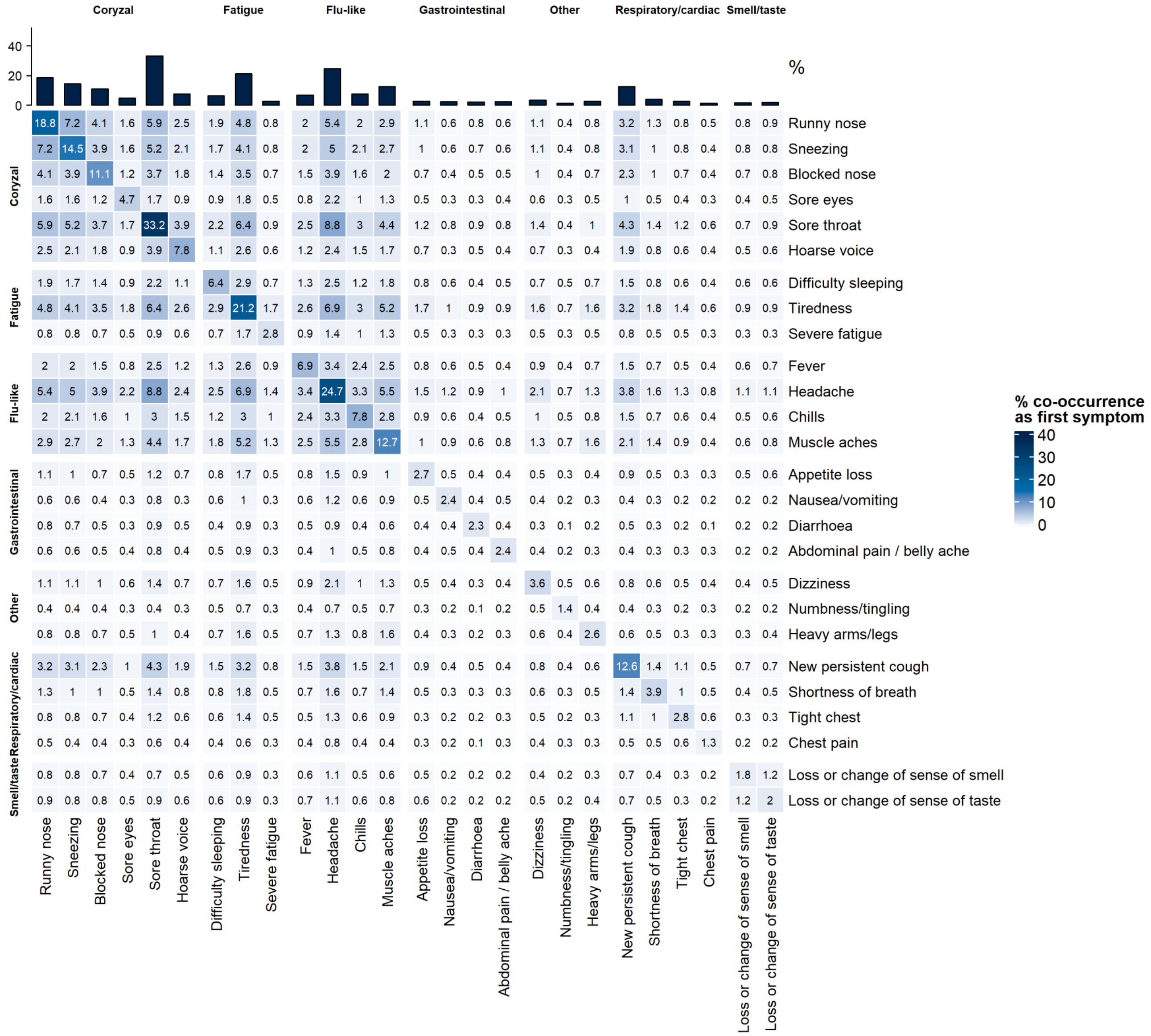

Supplementary Figure 6 Scatter plot showing N-gene Ct values among symptomatic swab-positive individuals plotted against time (in days) since symptom onset, for BA.1 and BA.2. For each variant, a linear regression model is fitted with Ct value as the dependent variable and days since symptom onset (between 1 and 11 or more). Pearson's R value shown, with a p-value derived from a two-sided test with H_0: r=0. For both variants there is a positive association between time since symptom onset and Ct value.

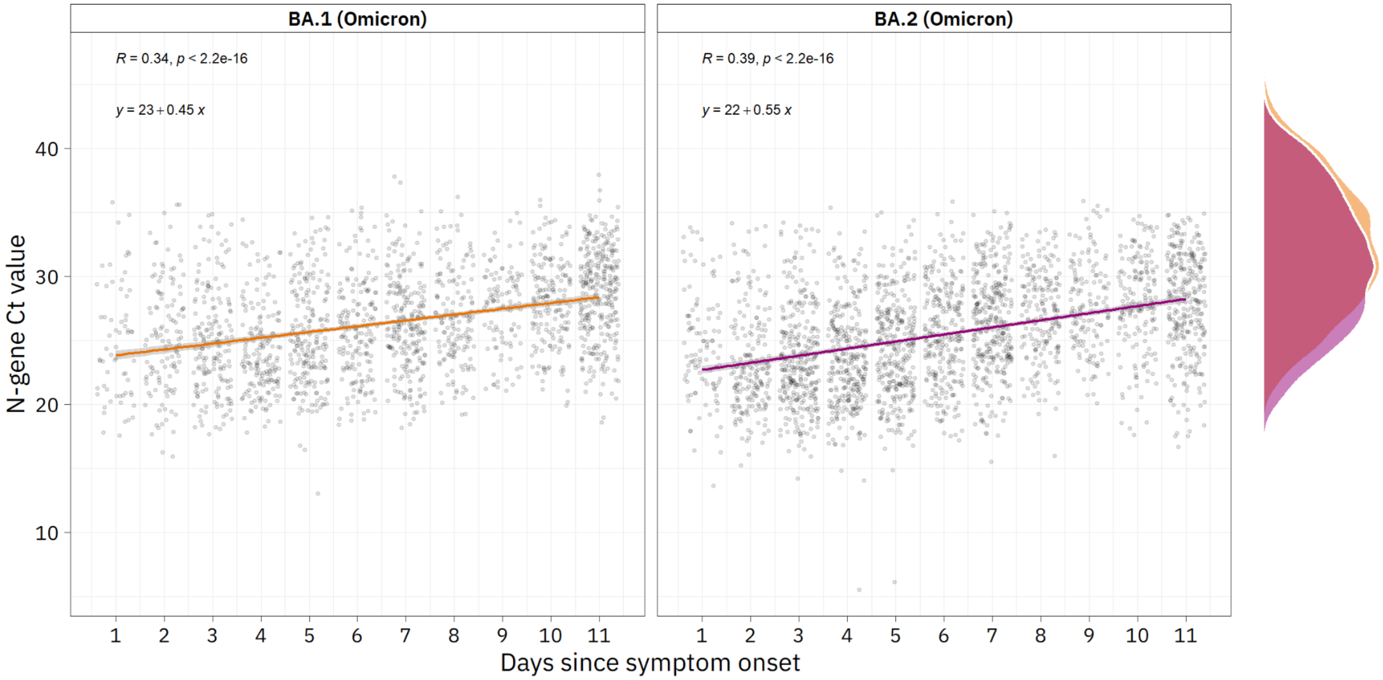

Supplementary Figure 7 Panel plot showing time since symptom onset across different variant phases. (A) shows the distribution of reported days-since-symptom-onset among N=13,134 PCR positives for the five variants. (B) shows the mean number of symptoms reported at each day after symptom onset, among N=13,134 PCR-positives for each of the five variants, plus 95% confidence intervals. Each variant is shown to have a similar symptom count trajectory, with symptom count peaking between 6 and 9 days after symptom onset. In the Wild-type, Alpha and Delta waves, respondents tended to report symptoms having started longer ago than in the Omicron waves.
(C) Presents the univariable analysis shown in Figure 2 among N=1,542,510 participants, additionally adjusted for time since symptom onset (as a categorical variable, 0–4, 5–10, 11+ days). Error bars show 95% confidence intervals. Odds ratios are attenuated but the patterns are largely consistent with the main analysis (Figure 2).

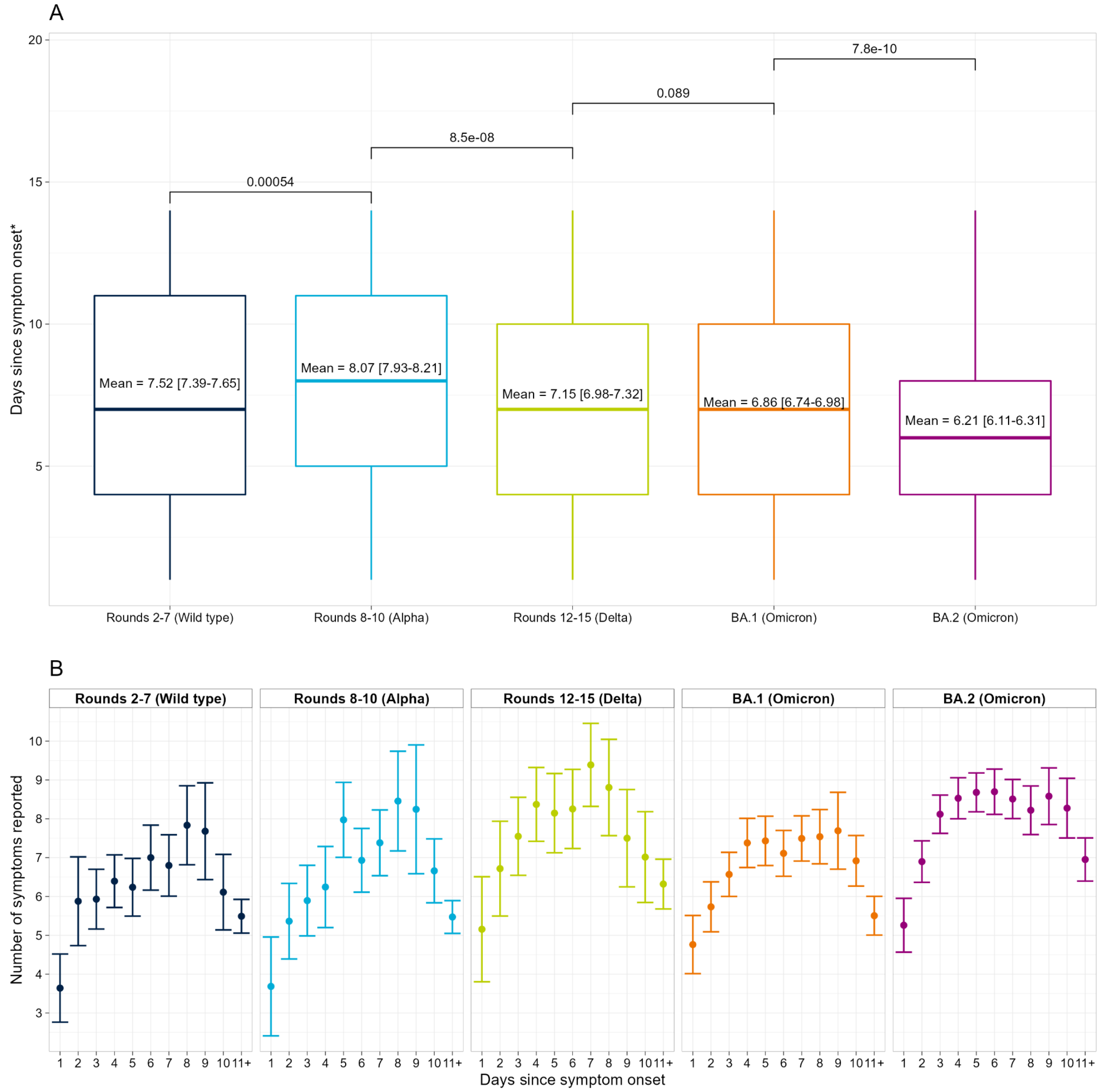

*Note: for those who reported "11+" days in response to the 'how long ago did your symptoms start?' question, we inferred the distribution of symptom durations using the estimates of PCR positivity duration (mean ~14 days) from Kojima *et al* (Kojima, N., Roshani, A. & Klausner, J. D. Duration of COVID-19 PCR positivity for Omicron vs earlier variants. *J. Clin. Virol* **2**, 100085 (2022)).

C

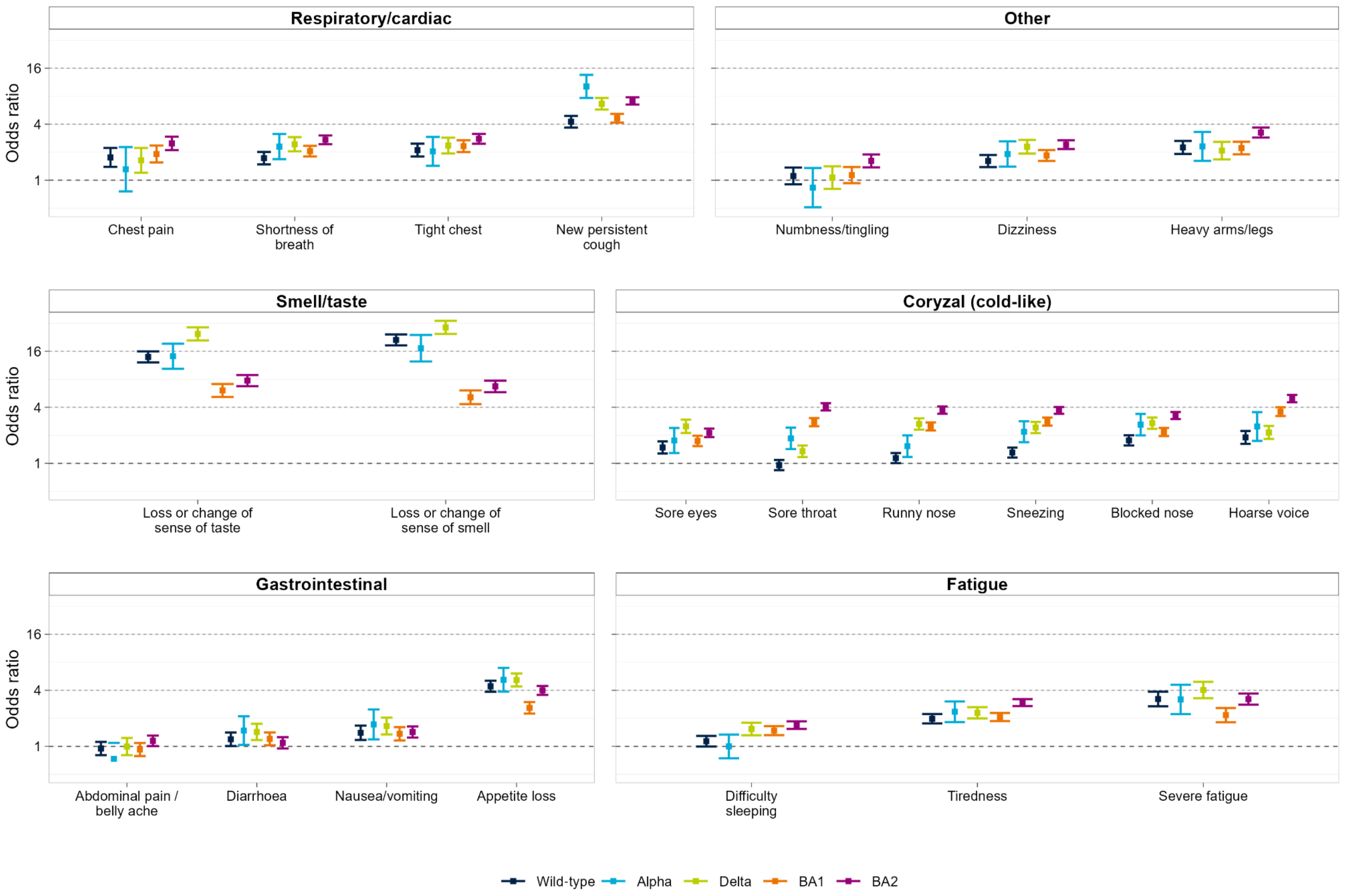

Respiratory/cardiac
Other
Odds ratio
16
4
1
Chest pain
Shortness of breath
Tight chest
New persistent cough
Numbness/tingling
Dizziness
Heavy arms/legs
Smell/taste
Coryzal (cold-like)
Loss or change of sense of taste
Loss or change of sense of smell
Sore eyes
Sore throat
Runny nose
Sneezing
Blocked nose
Hoarse voice
Gastrointestinal
Fatigue
Abdominal pain / belly ache
Diarrhoea
Nausea/vomiting
Appetite loss
Difficulty sleeping
Tiredness
Severe fatigue
Wild-type
Alpha
Delta
BA1
BA2

Figure S8 A) Boxplot showing the distribution of N-gene Ct values by symptom status among PCR positives, including the participants who were excluded from the main analysis owing to missing symptom status ('NA' in the plot). Significance levels are reported for each pairwise comparison, using the Kruskal-Wallis test. Median Ct values are shown within boxplots. Those with unknown symptom status are shown to have lower Ct values than aysmptomatics, but higher Ct values than those with 'classic' COVID-19 symptoms (cough, fever, loss or change of smell or taste) or any other symptoms. B) Panel plot showing the distribution of symptom status, including those of unknown status, by round, and in addition the prevalence of PCR positivity, and 95% binomial confidence intervals, by symptom status by round, in N=1,797,914 participants (the study population before exclusions for missing symptom data). Those with unknown symptom status consistently have a PCR positivity rate in between those with COVID-19 symptoms and those with no symptoms. Plots therefore suggest that, as expected, the 'unknown' symptom status group contains a mixture of people with symptoms and without.

A

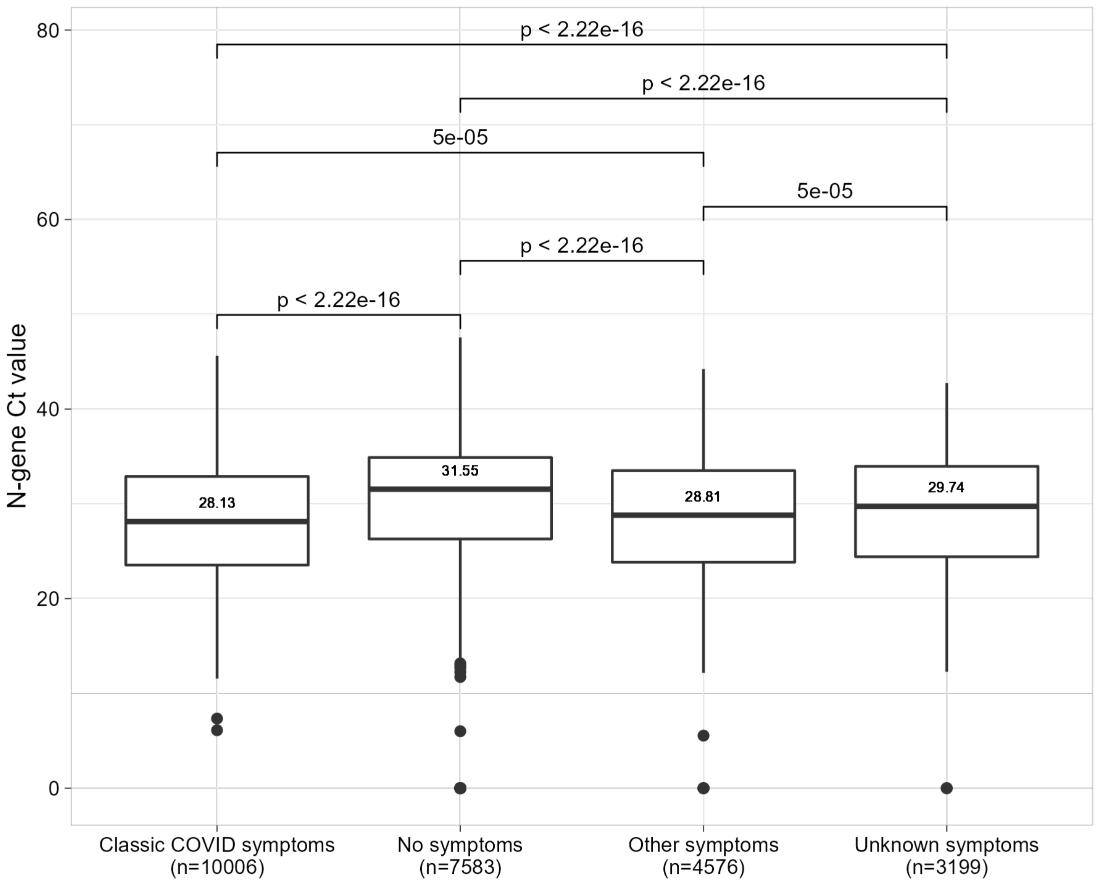

B

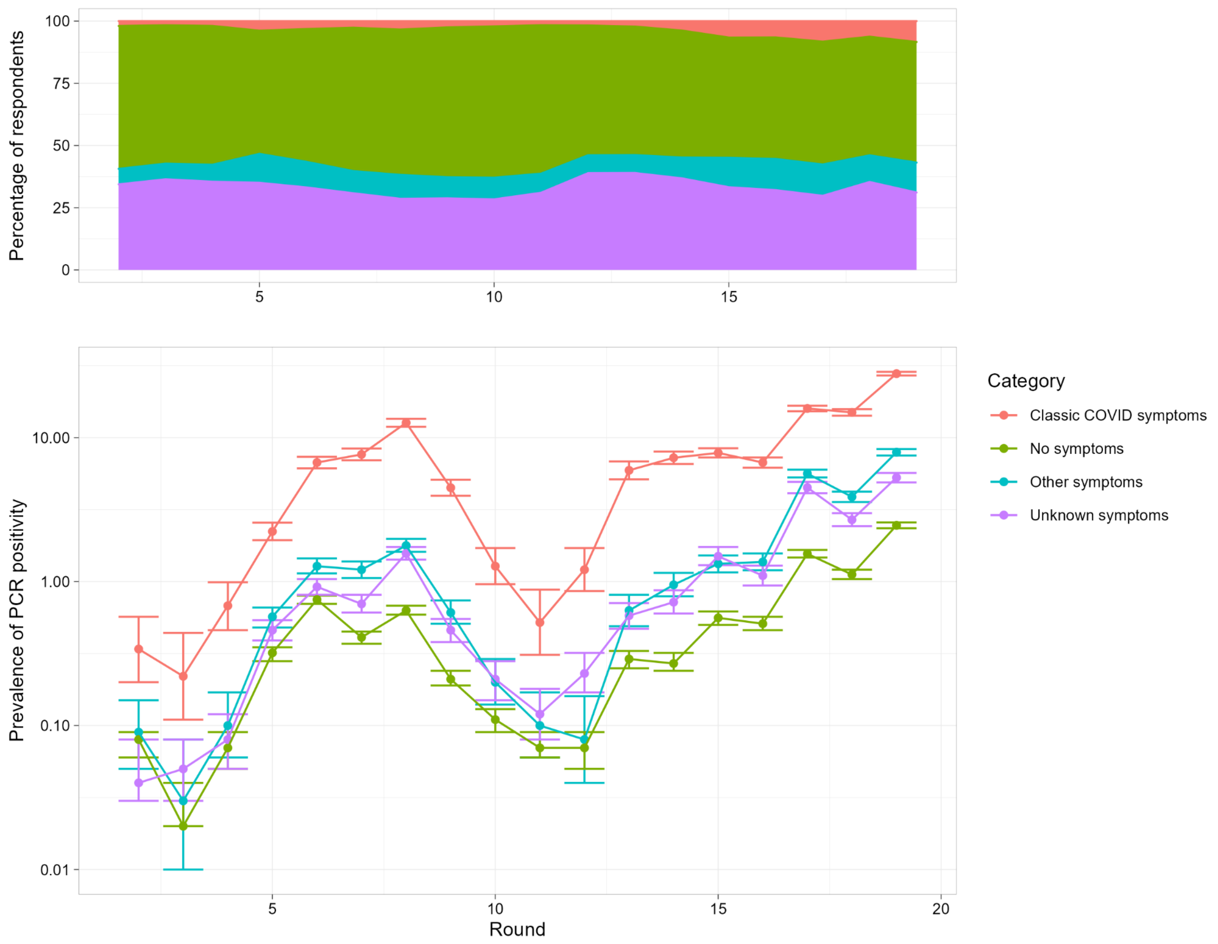

Percentage of respondents
100
75
50
25
0
5
10
15
Prevalence of PCR positivity
10.00
1.00
0.10
0.01
5
10
15
20
Round
Category
Classic COVID symptoms
No symptoms
Other symptoms
Unknown symptoms

Figure S9 Prevalence of any of 26 symptoms in N=1,525,062 PCR negative (top) vs N=17,448 PCR positive (bottom) individuals in each round of REACT-1 between June 2020 and March 2022. 95% binomial proportion confidence intervals are shown (owing to large sample size, these are very narrow in PCR negatives). Background symptom prevalence (ie prevalence among PCR negatives) peaks in round 19 (March 2022) at 20.94% [20.66, 21.22], with a low of 9.76% [9.59, 9.94] in round 2 (June 2020).

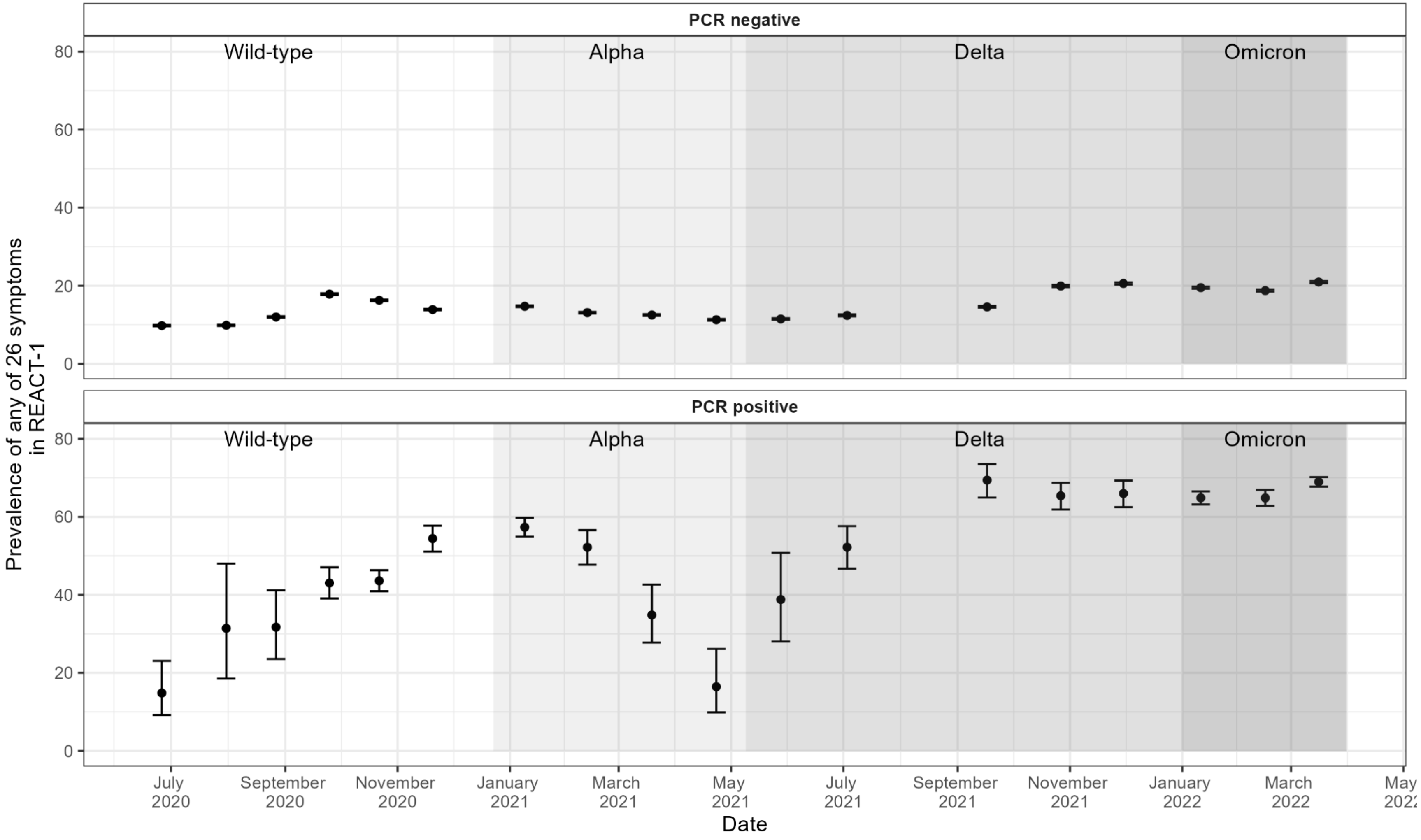

Supplementary methods

Survey symptom questions

In the past 4 weeks, that is since[date inserted], have you physically felt unwell or not quite right?
1. Yes
2. No

Have you/has your child had any of the following symptoms in the past 4 weeks, that is since [date inserted]? Please select all the symptoms you have had, whether or not you saw a doctor.

[select from list of symptoms]

Which, if any, of these symptoms have you had in the last 7 days, that is since [date inserted]?

[select from list of symptoms selected in previous question]

Thinking about the symptoms you had in the last 7 days, that is since [date inserted], which symptom started first?

[select from list of symptoms selected in previous question]

Data exclusions

266,847 participants were excluded because of missing symptom data. These participants were people who either skipped the top-level question 'have you felt unwell in the past month?' (n=266,361) or said that they had felt unwell but then did not tick any of the specific symptoms later in the survey, or declare 'none of these' (486 people). The symptom status of these people was therefore deemed to be unknown and they were removed from the analysis.

Model specifications

<u>Unpenalised logistic regression (main analysis, Figure 2, Table S3)</u>

Repeated for each of the 26 symptoms, plus a composite 'any of 26 symptoms' variable, for each of the variants:

y [PCR positive y/n] ~ *covariates* [age group, sex, vaccination count] + *symptom* [y/n]

<u>Unpenalised logistic regression (pooled analysis, Figure S2)</u>

For for each of the 26 symptoms, plus a composite 'any of 26 symptoms' variable:

y [PCR positive y/n] ~ *covariates* [age group, sex, vaccination count, calendar time] + *symptom* + variant + *symptom* [y/n]*variant

<u>Unpenalised logistic regression (severity modelling analysis, Table 1)</u>

y [Daily activities affected 'a lot' by symptoms/not] ~ *covariates* [Omicron BA.1/BA.2, age group, sex, vaccination count, Prior COVID-19, weeks since symptom onset, calendar time]*

**Covariates added incrementally in this order, and Odds Ratios reported for each additionally adjusted model*

<u>Unpenalised log-linear regression with Poisson errors (symptom count modelling analysis, Table S4)</u>

y [Symptom count] ~ *covariates* [Omicron BA.1/BA.2, age group, sex, vaccination count, Prior COVID-19, weeks since symptom onset, calendar time]*

**Covariates added incrementally in this order, and Odds Ratios reported for each additionally adjusted model*

<u>Penalised logistic regression (variable selection)</u>

Repeated for each variant x100 and variable selection proportions calculated:

y [PCR positive y/n] ~ *covariates* [age group, sex, vaccination count] + *symptoms* [each of 26 symptoms included as separate binary predictors]

The LASSO model minimises the model error subject to the constraint that the sum of absolute beta coefficients is less than a certain value (calibrated as part of the model fitting process). We imposed an additional constraint that no individual beta on the symptoms can take a value of less than zero.

Assessing model performance in variable selection

To quantify the gain in predictive accuracy conferred by each selected symptom, un-penalised logistic models were successively re-fit on 80% subsamples of the holdout data, adding each symptom in order of decreasing selection proportion, and evaluated on the remainder of the holdout data (20%). Age, sex and vaccination status were forced in as predictors in all models. For each set of predictors, the procedure was repeated 100 times with different splits of the holdout data. As in the variable selection, regression coefficients were constrained to non-negativity in the re-fit models.
