# Supplementary material for: Variant-specific symptoms of COVID-19 in a study of 1,542,510 adults in England
Source: Nat Commun. 2022 Nov 11;13:6856. doi: 10.1038/s41467-022-34244-2 (PMC9651890; doi:10.1038/s41467-022-34244-2)
Supplement: Supplementary file 3 — Reporting Summary [file 41467_2022_34244_MOESM3_ESM.pdf]

nature portfolio

Corresponding author(s): Prof Paul Elliott
Last updated by author(s): 13/10/22

Reporting Summary

Nature Portfolio wishes to improve the reproducibility of the work that we publish. This form provides structure for consistency and transparency in reporting. For further information on Nature Portfolio policies, see our Editorial Policies and the Editorial Policy Checklist.

Statistics

For all statistical analyses, confirm that the following items are present in the figure legend, table legend, main text, or Methods section.

n/a	Confirmed	
☐	☒	The exact sample size (*n*) for each experimental group/condition, given as a discrete number and unit of measurement
☐	☒	A statement on whether measurements were taken from distinct samples or whether the same sample was measured repeatedly
☐	☒	The statistical test(s) used AND whether they are one- or two-sided<br>*Only common tests should be described solely by name; describe more complex techniques in the Methods section.*
☐	☒	A description of all covariates tested
☒	☐	A description of any assumptions or corrections, such as tests of normality and adjustment for multiple comparisons
☐	☒	A full description of the statistical parameters including central tendency (e.g. means) or other basic estimates (e.g. regression coefficient) AND variation (e.g. standard deviation) or associated estimates of uncertainty (e.g. confidence intervals)
☐	☒	For null hypothesis testing, the test statistic (e.g. *F*, *t*, *r*) with confidence intervals, effect sizes, degrees of freedom and *P* value noted<br>*Give P values as exact values whenever suitable.*
☒	☐	For Bayesian analysis, information on the choice of priors and Markov chain Monte Carlo settings
☒	☐	For hierarchical and complex designs, identification of the appropriate level for tests and full reporting of outcomes
☒	☐	Estimates of effect sizes (e.g. Cohen's *d*, Pearson's *r*), indicating how they were calculated

Our web collection on statistics for biologists contains articles on many of the points above.

Software and code

Policy information about availability of computer code

Data collection	All data collection for the REACT study is captured with Questback (Spring 2020 installation).
Data analysis	All data were analysed using R v4.0.5 (2021).

For manuscripts utilizing custom algorithms or software that are central to the research but not yet described in published literature, software must be made available to editors and reviewers. We strongly encourage code deposition in a community repository (e.g. GitHub). See the Nature Portfolio guidelines for submitting code & software for further information.

Data

Policy information about availability of data

All manuscripts must include a data availability statement. This statement should provide the following information, where applicable:

- Accession codes, unique identifiers, or web links for publicly available datasets
- A description of any restrictions on data availability
- For clinical datasets or third party data, please ensure that the statement adheres to our policy

The original datasets generated or analysed, or both, during this study are not publicly available because of governance restrictions and the identifiable nature of the data. Requests for access to raw data should be addressed to the corresponding authors and will be answered within 12 weeks. Summary statistics, descriptive tables, and code from the current REACT-1 study are available at https://github.com/mrc-ide/reactidd/tree/master/inst/extdata/variant_symptom_profiling_paper. REACT-1 study materials are available for each round at

https://www.imperial.ac.uk/medicine/research-and-impact/groups/react-study/for-researchers/react-1-study-materials/
Sequence read data are available without restriction from the European Nucleotide Archive at https://www.ebi.ac.uk/ena/browser/view/PRJEB37886, and consensus genome sequences are available from the Global initiative on sharing all influenza data (GISAID)[26]. GISAID accession numbers for all sequences in the REACT1 study have been published in Eales et al[27] (supplementary data 1).

Human research participants

Policy information about studies involving human research participants and Sex and Gender in Research.

Reporting on sex and gender	Sex assigned at birth was determined by NHS records and accordingly 'sex' is used throughout the paper.
Population characteristics	Full population (N) = 1,542,510<br>Male = 670,270; Female = 872,240<br>Of which 17,448 tested PCR positive at the time of the survey
Recruitment	Every 4-6 weeks between May 2020 and March 2022, recruitment letters were sent to a random, nationally representative sample of people aged 5 years and over in England, using the National Health Service patient register. Participants then obtained self-administered throat and nasal swabs for SARS-CoV-2 PCR testing and completed an on line or telephone questionnaire which included questions on demographic variables, behaviour, and recent symptoms. Questionnaires for each of the 19 completed rounds since May 2020 are available on the study website (https://www.imperial.ac.uk/medicine/research-and-impact/groups/react-study/for-researchers/react-1-study-materials/). Between 95,000 and 175,000 viable swabs and valid responses were gathered each round, with respondents unaware of their test result at the time of their response.<br><br>While participants were blinded to the results of their PCR swab test at the tine of the test, there is the possibility of selection biases affecting recruitment: participants who had been exposed to COVID-19 may have been motivated to take the test to discover their own infection status, which would inflate PCR positivity estimates, especially in earlier rounds when testing was not widely available.
Ethics oversight	South Central-Berkshire B Research Ethics Committee (IRAS ID: 283787)

Note that full information on the approval of the study protocol must also be provided in the manuscript.

Field-specific reporting

Please select the one below that is the best fit for your research. If you are not sure, read the appropriate sections before making your selection.

☒ Life sciences ☐ Behavioural & social sciences ☐ Ecological, evolutionary & environmental sciences

For a reference copy of the document with all sections, see nature.com/documents/nr-reporting-summary-flat.pdf

Life sciences study design

All studies must disclose on these points even when the disclosure is negative.

Sample size	Full sample size = 1,542,510, across 15 rounds of REACT-1. The REACT-1 study is powered conservatively to give information on every LTLA in England (n=315), in each round, under the assumption that prevalence in each Local Authority is independent. Full information on sample size calculation and rationale is available in the protocol paper: https://wellcomeopenresearch.org/articles/5-200/v2.
Data exclusions	A total of 266,847 participants were excluded because of missing symptom data, and 38 were excluded because of missing age or sex data resulting in a final study population, after exclusions, of 1,542,510 participants.<br>The participants who were excluded because of missing symptom data were people who either skipped the top-level question 'have you felt unwell in the past month?' (n=266,361) or said that they had felt unwell but then did not tick any of the specific symptoms later in the survey, or declare 'none of these' (486 people). The symptom status of these people was therefore deemed to be unknown and they were removed from the analysis.
Replication	Replication analysis was not directly undertaken, although multivariable model performance was evaluated on a holdout data set.<br>We conducted a set of sensitivity analyses using different model specifications and data aggregations (notably pooled analysis to replicate the odds ratios from the variant-stratified logistic regression, and the matched analyses to complement the BA.1/BA.2 symptom comparison) and found the results consistent with our primary analyses.<br>To aid reproducibility, all scripts for analyses are publicly available and methods could be applied to other similar data sets to verify findings.
Randomization	Randomisation not applicable to a cross-sectional study design.
Blinding	Participants were blinded to the results of their PCR test at the time of the survey (and were notified afterwards if testing positive). Other survey data were reported retrospectively. As this was a cross-sectional study design with no grouping, no blinding was or could be applied to the researchers.

Reporting for specific materials, systems and methods

We require information from authors about some types of materials, experimental systems and methods used in many studies. Here, indicate whether each material, system or method listed is relevant to your study. If you are not sure if a list item applies to your research, read the appropriate section before selecting a response.

Materials & experimental systems

n/a	Involved in the study
☒	☐ Antibodies
☒	☐ Eukaryotic cell lines
☒	☐ Palaeontology and archaeology
☒	☐ Animals and other organisms
☐	☒ Clinical data
☒	☐ Dual use research of concern

Methods

n/a	Involved in the study
☒	☐ ChIP-seq
☒	☐ Flow cytometry
☒	☐ MRI-based neuroimaging

Clinical data

Policy information about clinical studies

All manuscripts should comply with the ICMJE guidelines for publication of clinical research and a completed CONSORT checklist must be included with all submissions.

Clinical trial registration	South Central-Berkshire B Research Ethics Committee (IRAS ID: 283787)
Study protocol	https://wellcomeopenresearch.org/articles/5-200/v2
Data collection	Participants obtained self-administered throat and nasal swabs for SARS-CoV-2 PCR testing, which were collected by courier, or, in later rounds, dispatched by priority coronavirus postal service. Participants also completed an on line or telephone questionnaire which included questions on demographic variables, behaviour, and recent symptoms.
Outcomes	PCR positivity for SARS-CoV-2 virus in self-administered throat and nasal swabs.
